# Trajectories of health-related quality of life among people with a physical disability and/or chronic disease during and after rehabilitation: a longitudinal cohort study

**DOI:** 10.1007/s11136-020-02647-7

**Published:** 2020-09-28

**Authors:** B. L. Seves, F. Hoekstra, F. J. Hettinga, R. Dekker, L. H. V. van der Woude, T. Hoekstra

**Affiliations:** 1 Center for Human Movement Sciences, University Medical Center Groningen, University of Groningen, Groningen, The Netherlands; 2 School of Health and Exercise Sciences, University of British Columbia Okanagan, Kelowna, BC Canada; 3 Department of Sport, Exercise and Rehabilitation, Northumbria University, Newcastle, UK; 4 Department of Rehabilitation Medicine, University Medical Center Groningen, University of Groningen, Groningen, The Netherlands; 5 Department of Health Sciences and Amsterdam Public Health Research Institute, Vrije Universiteit Amsterdam, Amsterdam, The Netherlands

**Keywords:** Quality of life, Active lifestyle, Health promotion, Rehabilitation, Latent class growth (mixture) models, Activity pacing

## Abstract

**Purpose:**

To identify Health-related Quality of Life (HR-QoL) trajectories in a large heterogeneous cohort of people with a physical disability and/or chronic disease during and after rehabilitation and to determine which factors before discharge are associated with longitudinal trajectory membership.

**Methods:**

A total of 1100 people with a physical disability and/or chronic disease were included from the longitudinal cohort study Rehabilitation, Sports and Active lifestyle. All participants participated in a physical activity promotion programme in Dutch rehabilitation care. HR-QoL was assessed using the RAND-12 Health Status Inventory questionnaire at baseline (T0: 3–6 weeks before discharge) and at 14 (T1), 33 (T2) and 52 (T3) weeks after discharge from rehabilitation. A data-driven approach using Latent Class Growth Mixture modelling was used to determine HR-QoL trajectories. Multiple binomial multivariable logistic regression analyses were used to determine person-, disease- and lifestyle-related factors associated with trajectory membership.

**Results:**

Three HR-QoL trajectories were identified: moderate (*N* = 635), high (*N* = 429) and recovery (*N* = 36). Trajectory membership was associated with person-related factors (age and body mass index), disease-related factors (perceived fatigue, perceived pain and acceptance of the disease) and one lifestyle-related factor (alcohol consumption) before discharge from rehabilitation.

**Conclusions:**

Most of the people who participated in a physical activity promotion programme obtained a relatively stable but moderate HR-QoL. The identified HR-QoL trajectories among our heterogeneous cohort are disease-overarching. Our findings suggest that people in rehabilitation may benefit from person-centred advice on management of fatigue and pain (e.g. activity pacing) and the acceptance of the disability.

**Electronic supplementary material:**

The online version of this article (10.1007/s11136-020-02647-7) contains supplementary material, which is available to authorized users.

## Introduction

Improving health-related quality of life (HR-QoL) is one of the key objectives in today’s rehabilitation practice. When evaluating rehabilitation treatments, interventions taking place in rehabilitation practice and policy in health care, HR-QoL is often used as an outcome measure [1, 2]. In people with a physical disability and/or chronic disease, HR-QoL during rehabilitation is lower than in the non-disabled population [3]. More importantly, after rehabilitation, low levels of HR-QoL are commonly reported in people with a physical disability and/or chronic disease [4–6], and HR-QoL is poorer compared to a healthy reference population [7]. Low levels of HR-QoL are associated with secondary health conditions (e.g. fatigue, pain, obesity and cardiovascular diseases), whereby preventing secondary health conditions among this target population is an important step towards sustainable health [8] and healthy ageing. Furthermore, low levels of HR-QoL are associated with inactivity and sedentary behaviour in healthy adults [9, 10]. Also, previous literature found that physical activity is positively associated with all components of HR-QoL, except for mental health in people after rehabilitation [7]. Physical activity promotion programmes in rehabilitation care could have positive impact on improving HR-QoL by reducing secondary health conditions during but also after treatment has finished [4, 11, 12].

According to the literature, there is large heterogeneity in HR-QoL development among people with disabilities [7]. Therefore, investigating HR-QoL by looking at average levels within the sample is not as useful as by investigating subgroups with distinct developmental trajectories of HR-QoL. Previous studies already identified several trajectories of HR-QoL in people during or after rehabilitation from breast cancer or stroke, which were related to the proposed characteristic trajectories of level of dysfunction: high, recovery, decline and low HR-QoL [13–15].

Cross-sectional research into the determinants of HR-QoL has found that personal factors (e.g. age and gender) are associated with HR-QoL in people with heart diseases [16] and in aneurysmal subarachnoid haemorrhage (SAH) survivors [17]. Psychosocial factors (e.g. self-efficacy, acceptance, passive coping) are associated with longitudinal HR-QoL in breast cancer survivors [13], in people post stroke [18] and in SAH survivors [17]. Psychological factors (e.g. depression, anxiety and fatigue) predict longitudinal trajectory membership of HR-QoL trajectories in people post stroke [14] and in SAH survivors [19] and predict cross-sectional HR-QoL in people with renal cell carcinoma [20]. Disease-related factors such as disease awareness in people after traumatic brain injury [21] and having comorbidities in people with renal cell carcinoma [20] were associated with, respectively, cross-sectional and longitudinal HR-QoL.

Most rehabilitation treatments or interventions to promote physical activity have not been evaluated for effectiveness on sustainable HR-QoL after rehabilitation treatment [2, 22]. So far, very little attention has been paid to a disease-overarching mechanism in the heterogeneous course of HR-QoL after rehabilitation. Previous research on HR-QoL development usually focussed on specific disease populations. The current longitudinal study provides an important opportunity to advance the understanding of the course of HR-QoL after rehabilitation, by undertaking a disease-overarching prospective analysis of HR-QoL. In addition, more insight into relevant determinants, such as person-, disease- and lifestyle-related factors is needed to identify vulnerable people with a physical disability and/or chronic disease at risk to experience a reduced HR-QoL after discharge already in the early stages of rehabilitation. These determinants can be non-modifiable (e.g. gender, age, severity of the disability) or modifiable (e.g. physical activity behaviour, acceptance of the disability, the use of tobacco and alcohol). Modifiable factors should be targeted by rehabilitation professionals, to improve patients’ HR-QoL. The findings of this study may support the need for more person-centred care to help people to obtain and maintain sustainable high levels of HR-QoL after rehabilitation.

Therefore, the purposes of this study were (1) to identify trajectories of HR-QoL up to 1 year after discharge from rehabilitation in people with a physical disability and/or chronic disease and (2) to determine person-, disease- and lifestyle-related factors before discharge from rehabilitation that are associated with longitudinal trajectory membership.

## Methods

### Context

The current study is part of the multicentre longitudinal cohort study Rehabilitation, Sports and Active lifestyle (ReSpAct) that was initiated to evaluate the nationwide programme Rehabilitation, Sports and Exercise (RSE; Dutch: ‘Revalidatie, Sport en Bewegen’) [23, 24]. The RSE programme has been implemented in eighteen rehabilitation institutions in the Netherlands (twelve rehabilitation centres and six rehabilitation departments of hospitals). The RSE programme aims to stimulate an active lifestyle during the rehabilitation period and to guide people with a physical disability and/or chronic disease in maintaining a physically active lifestyle in the home setting after discharge from rehabilitation [23, 24]. Participants of the RSE programme were referred to a sports counselling counter 3 to 6 weeks before discharge from rehabilitation for a face-to-face consultation with a sports counsellor, followed by four telephone-based counselling sessions up to 13 weeks after discharge from rehabilitation [23, 24]. All sessions were based on motivational interviewing [25] (see Online Resource 2 for a schematic overview of the RSE programme and the ReSpAct study).

Participants were included in the ReSpAct study from May 2013 to August 2015. Participants were monitored with questionnaires at given regular measurement times: at baseline (T0: 3–6 weeks before discharge) and 14 (T1), 33 (T2) and 52 (T3) weeks after discharge from rehabilitation (Online Resource 2). The study was approved by the ethics committee of the Center for Human Movement Sciences of the University Medical Center Groningen (reference: ECB/2013.02.28_1). All participants voluntarily participated after signing an informed consent.

### Study population

Inclusion criteria were: (1) being at least 18 years of age, (2) having a chronic disease or physical disability (e.g. stroke, heart failure, Parkinson’s disease, spinal cord injury), (3) receiving inpatient or outpatient rehabilitation care or treatment at one of the participating rehabilitation departments or institutions, (4) participating in the RSE programme [24] and (5) filling in the RAND-12 Health Status Inventory (RAND-12) at two or more measurement occasions. Participants were excluded if they were not able to complete the questionnaires, even with help, or were participating in another physical activity stimulation programme.

### HR-QoL

HR-QoL was assessed by using the self-reported RAND-12 questionnaire [26], an adapted, abbreviated version of the RAND-36 Health Status Inventory (RAND-36) [27]. The RAND-12 contains at least one item from each of the eight subscales of the RAND-36, so that it adequately represents the wide range of relevant aspects of health status [28]. Six items of the RAND-12 contribute to the physical health composite (how health limits a person in activities, or how a person’s physical health causes problems with work or other activities) and six other items contribute to the mental health composite (how a person feels and how a person’s mental health causes problems with work or other activities) [27, 28]. All twelve items contribute to the general health composite, which represents all relevant aspects of health status [28]. We used an age-corrected general health composite score for this study [27]. A higher score on the RAND-12 indicated better HR-QoL. Because the RAND-12 only contains twelve items of the RAND-36 (range 0–100), scores on the RAND-12 range from 0 to 65. We found good reliability (internal consistency) of the RAND-12 based on the study sample at T0 (Cronbach’s *α* = 0.85, *N* = 974), at T1 (Cronbach’s *α* = 0.87, *N* = 957), at T2 (Cronbach’s *α*  = 0.88, *N* = 861) and at T3 (Cronbach’s *α* = 0.88, *N* = 780). Previous literature supports acceptable construct validity and test–retest reliability of the RAND-12 in among others clinical populations [28, 29].

### Person-, disease- and lifestyle-related factors

All independent variables were measured at baseline (T0: 3–6 weeks before discharge). Person-related factors included gender, age, body mass index (BMI) and level of education, which was dichotomized into low (up to completed secondary education) and high (completed applied University or higher) to make it internationally comparable.

Disease-related factors included the type of disease divided into eight categories: musculoskeletal disease, amputation, brain disorder (e.g. stroke or other non-congenital brain defects), spinal cord injury, other neurologic disease, organ disease, chronic pain and other diseases. Also, disease-related factors included the number of comorbidities dichotomized into no comorbidities and one or more comorbidities, because this variable included all diseases and disabilities reported by a participant. The level of acceptance of the disability or disease was assessed on a four-point Likert scale (1–4, no acceptance to complete acceptance), with a higher score indicating better acceptance of the disability or disease. The level of acceptance was dichotomized into no (no or little acceptance) and yes (acceptance to a large extent or completely), because when entering the level of acceptance as categorical variable in the logistic regression, we found that the odds ratios (ORs) did not linearly increased/decreased. Perceived fatigue was assessed with the 9-item Fatigue Severity Scale (FSS) [30], which is a valid and reliable questionnaire to determine the impact of perceived fatigue in clinical populations (in people with systematic lupus erythematosus *r*_validity_ = 0.81 and *r*_reliability_ = 0.89, and in people with multiple sclerosis *r*_validity_ = 0.47 and *r*_reliability_ = 0.81) [30–32]. The FSS score ranges from 1 to 7, with a higher score indicating more perceived fatigue [30]. We found good reliability (internal consistency) of the FSS based on the study sample at T0 (Cronbach’s *α* = 0.91, *N* = 1044). The FSS includes items like “Exercise brings on my fatigue.” and “I am easily fatigued” [30]. The level of perceived pain was assessed on a six-point Likert scale (1–6, from no pain to severe pain), with a higher score indicating more perceived pain. The level of pain was dichotomized into no (no to light pain: score 1–3) and yes (moderate to severe pain: score: 4–6), because when entering perceived pain as categorical variable in the logistic regression, we found that the ORs did not linearly increased/decreased. Also, too few people reported severe pain (perceived pain = 6).

Lifestyle-related factors included the dichotomous variables smoking and alcohol use (“Do you smoke currently?” and “Do you consume alcohol currently?”: yes or no). In addition, the total minutes of physical activity per week was assessed by using the Adapted Short Questionnaire to Assess Health-enhancing physical activity (Adapted-SQUASH), a 19-item self-reported recall questionnaire. In a previous study, the Adapted-SQUASH has been shown to be a sufficiently reliable (intraclass correlation coefficient = 0.76, *p* < 0.001) and valid—compared to the Actiheart activity monitor—(intraclass correlation coefficient = 0.22, *p* = 0.027) questionnaire to determine self-reported physical activity in a similar sample (people with a physical disability and/or chronic disease) [33]. The Adapted-SQUASH is pre-structured in four main domains outlining types and settings of activity: ‘commuting traffic’, ‘activities at work and school’, ‘household activities’ and ‘leisure time activities’ including ‘sports activities’ [34]. The SQUASH [34] was adapted to make the questionnaire more applicable for this population (Adapted-SQUASH), as described in the study protocol of the ReSpAct study [24]. First, the items ‘wheeling in a wheelchair’ and ‘handcycling’ were added in the domains ‘commuting activities and leisure time’ and ‘sports activities’. Second, the self-reported intensity of the activity was categorised in ‘light’, ‘moderate’ and ‘vigorous’, instead of ‘slow’, ‘moderate’ and ‘fast’. Third, a large range of adapted sports (e.g. wheelchair basketball/rugby/tennis) were included for the item ‘sports activities’. Lastly, in the examples of different sports ‘tennis’ was replaced by ‘(wheelchair) tennis’. Information on sports participation (yes/no) was obtained from the Adapted-SQUASH. If the participant reported to perform at least one sports activity per week, than they were coded as ‘yes’, if not as ‘no’.

### Statistical analysis

Analyses were conducted in a two-step approach. First, trajectories of HR-QoL during and after rehabilitation among participants with two or more valid measurements over time were identified using Latent Class Growth Mixture (LCGM) modelling with quadratic (assuming non-linear change over time), linear (assuming linear change over time) and latent class analyses (lca) models [35], using the Mplus software program 7.11. The choice for linear and quadratic models was made based on previous research [14], showing trajectories of HR-QoL to be both linear as well as quadratic (non-linear). Additionally, latent class analyses were conducted for descriptive purposes. These analyses gave us insight in the (heterogeneity of) patterns of change in HR-QoL without a priori assuming a trajectory shape. LCGM models are regression-based models that assume that individuals in the sample do not necessarily come from one underlying population but might come from multiple underlying (or latent) subpopulations. LCGM modelling aims to find the optimal number and characteristics of these subpopulations. Common, stepwise modelling strategies were applied [35], using the Guidelines for Reporting on Latent Trajectory Studies (GRoLTS) as well [36]. A one-class model was first determined, thus assuming one underlying population, and subsequently more classes were added one at a time and model fit indices were inspected. The optimal number of classes was determined according to the following model fit criteria: (1) a lower Bayesian Information Criterion (BIC), where a difference of 10 points lower is usually regarded as sufficient improvement [37], (2) a higher entropy (range from 0 to 1), a standardised measure of how accurately individuals’ trajectories are classified, where higher values indicate better classification [38, 39] and (3) average posterior probabilities of ≥ 0.80 [35]. The choice for the optimal number of classes was additionally made considering clinical interpretation (rejecting solutions that do not make clinical sense) and class size. Finally, individuals were classified into their most likely class based on their posterior probability.

Second, multiple binomial multivariable logistic regression analyses were performed to assess associations between the previously described person-, disease- and lifestyle-related factors and trajectory membership using version 24 of the Statistical Package for the Social Science (SPSS). The outcome of the LCGM modelling, the nominal variable of trajectory membership, was used as dependent variable.

Independent variables at baseline were all entered block wise (block 1: person-related factors, block 2: disease-related factors and block 3: lifestyle-related factors) in multivariable models. Descriptive statistics of these variables were analysed at baseline. Assumptions of normality and linearity were checked. The continuous independent variables age, BMI, fatigue, and physical activity/week were standardised. Results of the multiple binomial multivariable logistic regression analyses are presented as odds ratio (OR) and corresponding 95% confidence interval (CI). Because three comparisons between two trajectories were needed to compare all HR-QoL trajectories, a Bonferroni-corrected *p*-value, to correct for multiple testing, of 0.017 (0.05/3 = 0.017) was used to give a 95% probability of correctly concluding not to reject the null hypothesis [40].

To facilitate transparency and reproducibility, additional information is available on: (a) the dataset of the HR-QoL (Online Resource 1) and (b) the Mplus syntax of the LCGM modelling and the SPSS syntax of the multiple binomial multivariable logistic regression analyses (Online Resource 2).

## Results

### Characteristics of participants

In total 1100 participants were included in this study. Participants had an average age of 51.0 ± 13.5 years and 52.0% were female. The three most common disease groups were brain disorder (26.0%, *N* = 286), musculoskeletal disease (18.1%, *N* = 199) and chronic pain (15.6%, *N* = 172) (Table 1).

**Table 1:** Participants’ descriptive statistics at baseline for participants included (*N* = 1100) and excluded (*N* = 617) in the latent class growth mixture modelling analyses

Characteristic	Included in LCGMM	Excluded for LCGMM
	Mean ± SD or % (*N*)	Mean ± SD or % (*N*)
Personal-related factors		
Gender (% female)	52.0 (572)	57.8 (358)*
Age in years	51.0 ± 13.5	47.8 ± 13.9**
Body mass index (kg/m^2^)	27.2 ± 5.5	27.6 ± 6.2
Education level (% high)^a^	24.5 (270)	11.5 (71)*
Living situation (% independent)	88.7 (976)	53.0 (328)*
Disease-related factors		
Disease group		
Brain disorders	26.0 (286)	27.1 (168)
Musculoskeletal disease	18.1 (199)	19.2 (119)
Chronic pain	15.6 (172)	17.8 (110)
Neurologic disease	15.5 (171)	12.1 (75)
Organ disease	12.0 (132)	10.7 (66)
Amputation	4.5 (50)	4.4 (27)
Other symptoms	4.0 (44)	3.1 (19)
Spinal cord injury	2.8 (31)	4.4 (27)
Acceptance (% yes)	54.3 (597)	28.4 (176)*
Comorbidities (% yes)	41.3 (454)	28.1 (174)
Fatigue (FSS score)	4.3 ± 1.5	4.5 ± 1.5*
Pain (% yes)	46.2 (508)	25.7 (159)
Lifestyle-related factors		
Smoking (% yes)	16.4 (180)	13.7 (85)*
Alcohol use (% yes)	39.1 (430)	18.6 (115)
Total minutes of PA/week	1081.1 ± 919.5	1120.8 ± 966.8
Sports participation (% yes)	54.5 (600)	45.6 (282)
Institutional level		
Treatment form (% outpatient)^c^	90.4 (994)	89.0 (551)
Treatment context (% hospital)	28.1 (309)	26.2 (162)
Amount of physical activity counselling moments after rehabilitation^d^	2.6 ± 1.4	2.1 ± 1.5*
Health-related quality of life (RAND-12)		
Mental health composite	40.3 ± 9.4	38.5 ± 9.3*
Physical health composite	36.2 ± 10.3	33.6 ± 9.4**
General health composite	37.2 ± 9.3	34.7 ± 8.8**

*SD* standard deviation, *N* number of participants, *LCGMM* latent class growth mixture modelling, *FSS* Fatigue Severity Scale, *PA* Physical activity

^a^Completed applied University or higher

^b^Percentage of participants with one or more comorbidities

^c^Treatment form includes outpatient and inpatient

^d^Participants in the Rehabilitation, Sports and Exercise programme received four telephone-based counselling sessions with a sports counsellor

*and **The characteristic is significantly different (*p < 0.05, **p < 0.01) between the participants included and excluded for the LCGMM based on independent sample t-tests for continuous variables and based on Chi-square tests for categorical variables

Based on descriptive characteristics at baseline (Table 1), participants excluded for the LCGM modelling analyses were on average more often female, younger, lower educated, lived less independently, had worse acceptance of their disease, perceived more fatigue, smoked less, received less counselling moments and had lower levels of HR-QoL. Descriptive characteristics at baseline were missing of around 250 excluded participants, which might give skewed descriptive characteristics.

### HR-QoL trajectories

**Fig. 1:**
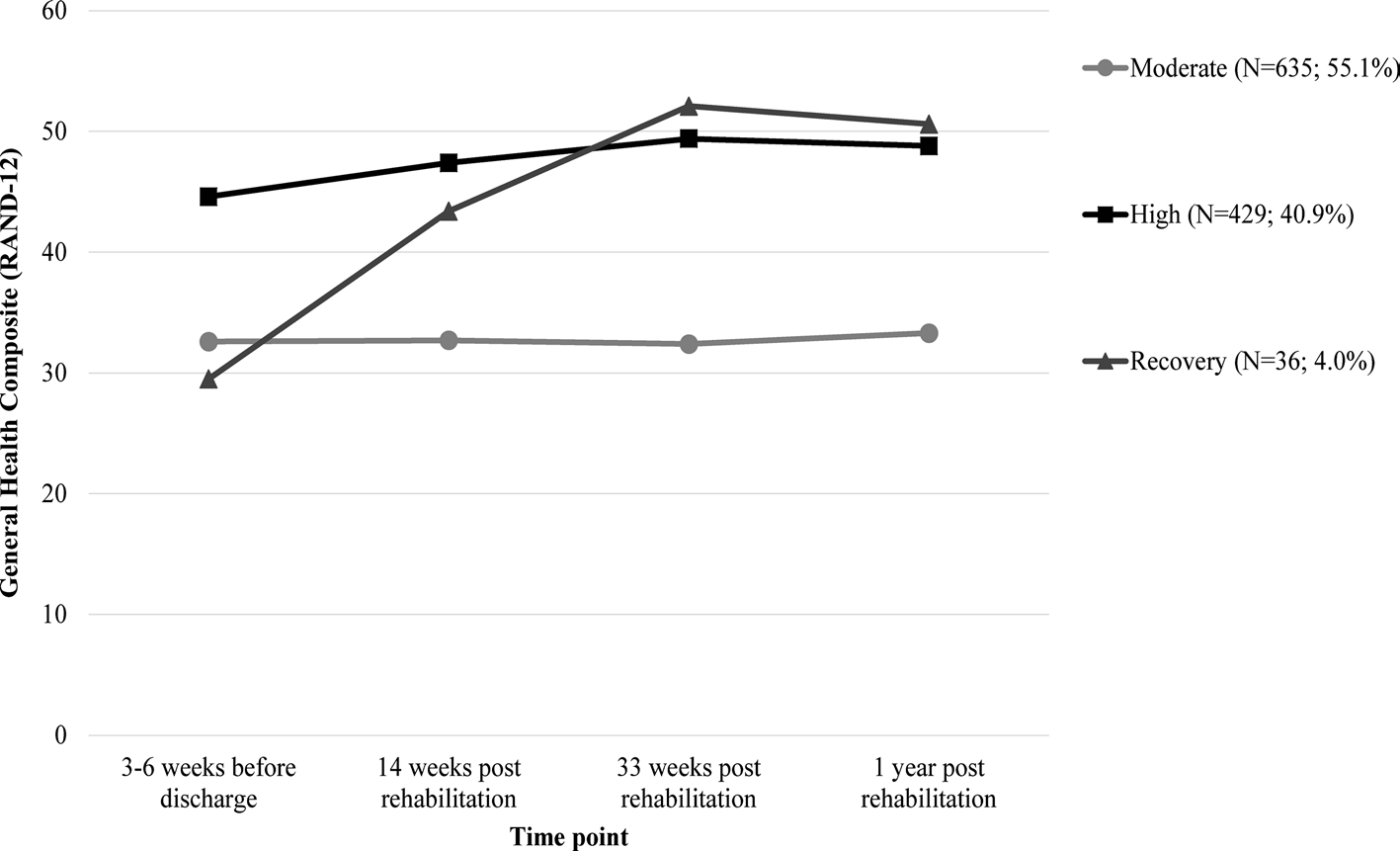
Three-trajectory model of HR-QoL (*N* = 1100), based on the general health composite (RAND-12)

The results of the fit indices for quadratic, linear and lca models with one to six trajectories of HR-QoL are presented in Table 2. Comparing these models with the model fit criteria alone proved to be complicated, as the model fit criteria were not always in agreement, which is a common finding in LCGM modelling [41]. After careful consideration, we chose the three-class quadratic model as the optimal model in this sample, although the average posterior probabilities were slightly below 0.80, indicating possibly less distinct trajectories and subsequent fuzzy classification, yet it avoids inclusion of an extremely small class, as is the case in the four-class and five-class quadratic models. The three-trajectory model consisted of two large and stable, but distinctly different trajectories: moderate (*N* = 635, 55.1%) and high (*N* = 429; 40.9%) trajectory. In addition, one smaller intermediate trajectory is provided, which increases between 3 and 6 weeks before discharge from rehabilitation and 33 weeks post rehabilitation and then stabilises (i.e. recovery) (*N* = 36; 4.0%) (Fig. 1).

**Table 2:** Fit indices for quadratic, linear and lca models with 1–6 trajectories of HR-QoL

Health-related quality of life									
Number of classes	BIC	Entropy	Average posterior probability (min–max)	Number of participants in each trajectory class					
				1	2	3	4	5	6
Quadratic analyses									
1	24,301.36	NA	1.0	1100					
2	24,227.49	.87	.90 (.83–.97)	1058	42				
** 3**	**24,198.33**	**.61**	**.79 (.76**–**.83)**	**36**	**635**	**429**			
4	24,201.32	.67	.83 (.77–.95)	2	640	42	416		
5	24,196.12	.69	.78 (.72–.83)	620	55	31	3	391	
6	24,204.48	.65	.78 (.64–.98)	53	595	2	34	370	46
Linear analyses									
1	24,254.81	NA	1.0	1100					
2	24,224.64	.98	.94 (.87–.99)	1093	7				
3	24,225.76	.64	.85 (.81–.90)	636	7	457			
4	24,228.39	.79	.84 (.80–.90)	993	71	7	30		
5	24,221.44	.63	.80 (.72–.90)	629	331	6	31	103	
6	24,237.72	.66	.78 (.71–.86)	5	320	32	126	615	2
lca analyses									
1	26,708.06	NA	1.0	1100					
2	25,283.89	.79	.94 (.94–.94)	603	497				
3	24,698.63	.81	.91 (.91–.91)	354	509	237			
4	24,504.05	.79	.88 (.86–.90)	229	119	414	338		
5	24,400.27	.78	.86 (.83–.91)	76	288	355	279	102	
6	24,367.06	.80	.85 (.76–.91)	79	16	352	286	265	102

In bold are the values of the chosen model

*BIC* Bayesian Information Criterion, *NA* not applicable, *lca* latent class analyses

Descriptive statistics of the mental, physical and general health composites for the three trajectories at each measurement time are presented in Table 3. Overall, mental health followed the same but higher course and physical health followed the same but lower course compared to general health. Supplementary figures are given in Online Resource 2, including estimated mean trajectories for each model, estimated means with individual trajectories for each latent class and the estimated with observed means for the final model. Although the plots with estimated means with individual trajectories for each latent class show large heterogeneity in individual trajectories of HR-QoL, all individual trajectories follow the same growth pattern over time for each latent class.

**Table 3:** Mental, physical and general HR-QoL for the three trajectories at baseline (T0: 3–6 weeks before discharge) and at 14 (T1), 33 (T2) and 52 (T3) weeks after discharge from rehabilitation

	T0	T1	T2	T3
	Mean ± SD	Mean ± SD	Mean ± SD	Mean ± SD
Mental health composite				
Moderate (*N* = 635)	36.2 ± 7.8	36.3 ± 7.7	35.9 ± 7.3	37.1 ± 8.4
High (*N* = 429)	46.9 ± 7.8	49.6 ± 7.5	51.2 ± 6.3	49.8 ± 7.9
Recovery (*N* = 36)	35.1 ± 7.5	46.7 ± 9.0	55.2 ± 6.2	53.9 ± 7.5
Physical health composite				
Moderate (*N* = 635)	32.0 ± 8.6	32.0 ± 8.6	31.9 ± 8.4	32.2 ± 8.9
High (*N* = 429)	43.1 ± 8.5	45.4 ± 8.0	47.3 ± 7.2	47.5 ± 7.5
Recovery (*N* = 36)	28.2 ± 10.6	41.1 ± 11.9	48.3 ± 8.2	46.9 ± 9.3
General health composite				
Moderate (*N* = 635)	32.6 ± 7.2	32.7 ± 7.0	32.4 ± 6.4	33.3 ± 7.6
High (*N* = 429)	44.6 ± 7.2	47.4 ± 6.9	49.4 ± 5.7	48.8 ± 7.0
Recovery (*N* = 36)	29.5 ± 7.1	43.4 ± 10.5	52.1 ± 6.0	50.6 ± 7.9

*SD* standard deviation, *N* Number of participants

Range: Mental health composite (13–66), Physical health composite (0–63), General health composite (6–65)

### Determinants of HR-QoL trajectories

Descriptive statistics of possible determinants before discharge from rehabilitation for the HR-QoL trajectories are presented in Table 4. Multiple binomial multivariable logistic regression analyses were performed to determine associations among the personal-, disease- and lifestyle-related factors before discharge from rehabilitation and the HR-QoL trajectories (Table 5).

**Table 4:** Person-, disease- and lifestyle-related factors at baseline for the three trajectories of HR-QoL

			Moderate (*N* = 635)	High (*N* = 429)	Recovery (*N* = 36)
			Mean ± SD or % (*N*)	Mean ± SD or % (*N*)	Mean ± SD or % (*N*)
Personal-related factors					
Gender (% female)			57.2 (363)	43.6 (187)	61.1 (22)
Age in years			50.3 ± 13.3	52.8 ± 13.5	42.8 ± 14.5
Body mass index (kg/m^2^)			27.9 ± 5.6	26.2 ± 5.0	27.4 ± 6.5
Education level (% high)^a^			21.3 (135)	28.9 (124)	30.6 (11)
Disease-related factors					
Disease group					
Musculoskeletal disease			20.0 (127)	13.5 (58)	38.9 (14)
Amputation			2.7 (17)	7.5 (32)	2.8 (1)
Brain disease			23.3 (148)	30.5 (131)	19.4 (7)
Neurologic disease			17.0 (108)	13.5 (58)	13.9 (5)
Spinal cord injury			2.4 (15)	3.7 (16)	0 (0)
Organ disease			9.6 (61)	15.9 (68)	8.3 (3)
Chronic pain			19.5 (124)	10.0 (43)	13.9 (5)
Other disease			3.8 (24)	4.4 (19)	2.8 (1)
Acceptance (% yes)			42.0 (267)	74.4 (319)	30.6 (11)
Comorbidities (% yes)			47.1 (299)	33.3 (143)	33.3 (12)
Fatigue (FSS score)			4.8 ± 1.3	3.6 ± 1.4	4.3 ± 1.3
Pain (% yes)			60.5 (384)	23.3 (100)	66.7 (24)
Lifestyle-related factors					
Smoking (% yes)			19.4 (123)	12.1 (52)	13.9 (5)
Alcohol use (% yes)			34.6 (220)	47.1 (202)	22.2 (8)
Total minutes of PA/week			1031.0 ± 884.9	1137.6 ± 956.8	1294.5 ± 1021.2
Sports participation (% yes)			52.3 (332)	58.5 (251)	47.2 (17)

^a^Completed applied University or higher

*SD* standard deviation, *N* number of participants, *PA* physical activity, *FSS* Fatigue Severity Scale

**Table 5:** Multiple binomial multivariable logistic regression analyses at baseline to distinguish between three pairs of three HR-QoL trajectories and the same comparisons with correction for general HR-QoL scores at baseline

			HR-QoL						HR-QoL, after correcting for baseline HR-QoL					
			Moderate (ref) vs. High		Recovery (ref) vs. Moderate		Recovery (ref) vs. High		Moderate (ref) vs. High		Recovery (ref) vs. Moderate		Recovery (ref) vs. High	
			OR (95% CI)	*p*	OR (95% CI)	*p*	OR (95% CI)	*p*	OR (95% CI)	*p*	OR (95% CI)	*p*	OR (95% CI)	*p*
HR-QoL at baseline			NA		NA		NA		**5.80 (4.10**–**8.21)**	** < .001**	2.05 (1.05–3.97)	.034	**45.18 (10.26**–**198.98)**	** < .001**
Personal-related factors														
Gender (female)			0.90 (0.62–1.31)	.584	1.47 (0.65–3.34)	.352	1.70 (0.62–4.67)	.308	0.93 (0.61–1.42)	.739	1.34 (0.59–3.04)	.489	2.24 (0.52–9.60)	.279
Age			1.27 (1.04–1.55)	.020	1.48 (0.94–2.33)	.094	**1.97 (1.18**–**3.29)**	**.010**	1.03 (0.82–1.29)	.815	1.22 (0.75–1.78)	.427	1.30 (0.67–2.52)	.438
Body Mass Index			**0.77 (0.64**–**0.94)**	**.009**	1.04 (0.69–1.56)	.849	0.80 (0.53–1.20)	.272	0.88 (0.71–1.09)	.249	1.09 (0.72–1.66)	.690	0.80 (0.46–1.39)	.423
Education (high)			1.41 (0.95–2.10)	.089	0.79 (0.32–1.95)	.602	0.70 (0.26–1.89)	.477	1.26 (0.81–1.96)	.312	0.76 (0.30–1.92)	.555	0.62 (0.15–2.68)	.526
Disease-related factors														
Disability (ref = musculoskeletal disease)				.549		.303		.874		.408		.265		.351
Amputation			1.86 (0.72–4.80)	.202	2.34 (0.23–24.04)	.473	2.63 (0.22–31.64)	.447	1.88 (0.63–5.58)	.257	2.54 (0.25–26.12)	.434	16.46 (0.62–437.36)	.094
Brain disorders			0.82 (0.46–1.47)	.505	5.10 (1.34–19.44)	.017	3.03 (0.72–12.67)	.129	0.78 (0.41–1.50)	.459	**5.23 (1.36**–**20.12)**	**.016**	6.03 (0.95–38.49)	.057
Neurologic disease			0.96 (0.51–1.80)	.886	2.49 (0.73–8.45)	.143	1.52 (0.39–6.04)	.548	0.97 (0.48–1.96)	.927	2.73 (0.81–9.20)	.106	2.17 (0.34–13.91)	.415
Spinal cord injury			2.07 (0.72–5.99)	.180	NA	.999	NA	.998	2.17 (0.69–6.83)	.186	NA	.999	NA	.998
Organ disease			1.02 (0.51–2.05)	.959	1.89 (0.41–8.81)	.418	1.19 (0.23–6.10)	.839	0.66 (0.30–1.47)	.312	1.74 (0.37–8.16)	.485	0.58 (0.07–5.18)	.628
Chronic pain			0.97 (0.52–1.82)	.926	4.02 (1.04–15.45)	.043	2.17 (0.46–10.23)	.328	0.78 (0.39–1.55)	.476	4.16 (1.06–16.29)	.040	2.27 (0.35–14.86)	.393
Other symptoms			0.75 (0.29–1.89)	.538	2.95 (0.31–28.27)	.348	1.47 (0.12–17.45)	.760	0.75 (0.26–2.15)	.594	3.09 (0.31–30.70)	.337	0.63 (0.04–9.89)	.740
Acceptance (yes)			**3.25 (2.25**–**4.68)**	** < .001**	1.65 (0.73–3.76)	.231	**5.09 (2.04**–**12.69)**	** < .001**	1.46 (0.96–2.23)	.077	1.14 (0.46–2.80)	.775	0.58 (0.15–2.34)	.447
Comorbidities (yes)			0.79 (0.55–1.16)	.228	1.55 (0.62–3.86)	.346	0.66 (0.23–1.91)	.443	0.90 (0.59–1.37)	.631	1.70 (0.70–4.32)	.265	1.49 (0.38–5.90)	.570
Fatigue (FSS score)			**0.47 (0.39**–**0.58)**	** < .001**	1.32 (0.87–2.00)	.200	0.67 (0.40–1.12)	.126	**0.69 (0.55**–**0.87)**	**.001**	1.54 (0.99–2.40)	.056	1.60 (0.74–3.44)	.229
Pain (yes)			**0.22 (0.15**–**0.33)**	** < .001**	1.59 (0.62–4.09)	.332	0.39 (0.14–1.98)	.072	**0.56 (0.35**–**0.88)**	**.011**	2.19 (0.80–5.94)	.125	2.16 (0.54–8.67)	.280
Lifestyle-related factors														
Smoking (yes)			0.58 (0.35–0.94)	.027	2.05 (0.64–6.59)	.226	1.00 (0.24–4.14)	.999	0.53 (0.30–0.94)	.028	2.02 (0.62–6.65)	.246	0.53 (0.10–2.73)	.450
Alcohol use (yes)			1.44 (1.01–2.05)	.043	3.05 (1.09–8.53)	.033	**4.60 (1.53**–**13.83)**	**.007**	1.41 (0.95–2.09)	.088	2.61 (0.92–7.42)	.071	2.62 (0.71–9.66)	.149
Total minutes of PA/week			1.21 (1.01–1.44)	.043	0.82 (0.57–1.19)	.303	1.12 (0.70–1.81)	.630	1.00 (0.81–1.24)	.976	0.77 (0.53–1.12)	.170	0.97 (0.56–1.70)	.921
Sports participation (yes)			1.11 (0.78–1.58)	.555	1.19 (0.53–2.66)	.669	1.28 (0.52–3.19)	.594	1.03 (0.70–1.53)	.871	1.17 (0.52–2.66)	.706	1.49 (0.47–4.70)	.495

Values in bold are significant (*p* < 0.017)

*HR-QoL* health-related quality of life, *OR* odds ratio, *CI* confidence interval, *ref* reference, *FSS* Fatigue Severity Scale, *PA* physical activity, *NA* not applicable

Compared with participants in the moderate HR-QoL trajectory (*N* = 635), participants with a higher BMI (OR 0.77, 95% CI 0.64–0.94), participants who perceive fatigue (OR 0.47, 95% CI 0.39–0.58) and/or participants who perceive pain (OR 0.22, 95% CI 0.15–0.33) are less likely to belong to the latent class with a high HR-QoL trajectory (*N* = 429), while participants who accept their physical disability and/or chronic disease (OR 3.25, 95% CI 2.25–4.68) are more likely to belong to the latent class with a high HR-QoL trajectory. Also compared to the moderate HR-QoL trajectory, based on the limits of the 95% CI which both lie above or below one (but not significant), participants who are older (OR 1.27, 95% CI 1.04–1.55), participants who drink alcohol (OR 1.44, 95% CI 1.01–2.05) and/or participants who are more physically active (OR 1.21, 95% CI 1.01–1.44) are more likely to belong to the latent class with a high HR-QoL trajectory, while participants who smoke (OR 0.58, 95% CI 0.35–0.94) are less likely to belong to this latent class.

There were no significant determinants before discharge to distinguish between the moderate HR-QoL (*N* = 635) and the recovery HR-QoL (*N* = 36) trajectories. But, based on the limits of the 95% CI which both lie above one (but not significant), participants who drink alcohol (OR 3.05, 95% CI 1.09–8.53) are more likely to belong to the latent class with a moderate HR-QoL trajectory, compared to the recovery HR-QoL trajectory.

A comparison of the recovery HR-QoL trajectory (*N* = 36) and the high HR-QoL trajectory (*N* = 429) showed that participants who are older (OR 1.97, 95% CI 1.18–3.29), participants who accept their physical disability and/or chronic disease (OR 5.09, 95% CI 2.04–12.69) and/or participants who drink alcohol (OR 4.60, 95% CI 1.53–13.83) are more likely to belong to the latent class with a high HR-QoL trajectory (*N* = 429).

Remarkably, gender, education level, type of disease, having comorbidities, level of physical activity and sports participation before discharge were not significant determinants to distinguish between trajectories of HR-QoL.

In addition, we checked whether the found significant determinants in the multiple binomial multivariable logistic regression analyses were still found after controlling for general HR-QoL scores at baseline (Table 5). HR-QoL scores at baseline were found to be significant determinants in the comparisons between the moderate and high HR-QoL trajectories (OR 5.86, 95% CI 4.14–8.30) and between the recovery and high HR-QoL trajectories (OR 45.24, 95% CI 10.26–199.47). When controlling for HR-QoL score at baseline, only perceived fatigue (OR 0.69, 95% CI 0.55–0.87) and perceived pain (OR 0.56, 95% CI 0.35–0.88) remain significant determinants when comparing the moderate and high HR-QoL trajectories (Table 5).

## Discussion

This study identified three distinct trajectories of HR-QoL up to 1 year after rehabilitation in a large heterogeneous cohort of people with a physical disability and/or chronic disease: moderate, high and recovery. The two large and stable trajectories of HR-QoL (moderate and high) among our sample are similar to the large HR-QoL trajectories identified in specific disease populations (e.g. stroke patients [14] and breast cancer survivors [13]), which might indicate that HR-QoL trajectories are not necessarily disease specific. However, we did not identify a decline in HR-QoL trajectory in our sample. Although a considerable group of our sample (40.9%) obtained stable high HR-QoL after participating in the physical activity promotion programme [23, 24], most of the sample (55.1%) did not.

This study determined which person-, disease- and lifestyle-related factors at discharge from rehabilitation are associated with trajectories of HR-QoL after rehabilitation. The following modifiable disease-related factors were determinants of trajectory membership: acceptance of the disability, perceived fatigue and pain before discharge from rehabilitation. These factors could be explored further for possibilities to modify the vulnerable trajectories into more favourable trajectories of HR-QoL. Acceptance of the disability before discharge from rehabilitation distinguished people in the high HR-QoL trajectory from people in both the moderate and the recovery HR-QoL trajectories. Van Mierlo et al. also found that the acceptance of the disability is a determinant for stable high HR-QoL compared with low HR-QoL in stroke patients [14]. This finding indicates the importance of paying attention to the acceptance of the disability during rehabilitation (e.g. focus on self-management and social/family support [42]), so that people are able to obtain and/or maintain high HR-QoL during and after rehabilitation.

In addition, less perceived fatigue and pain at discharge from rehabilitation strongly distinguishes people in the high HR-QoL trajectory from those in the moderate HR-QoL trajectory, even after controlling for baseline general HR-QoL scores. Fatigue is a distressing secondary health condition that is commonly reported in rehabilitation [43, 44]. Psychological/behavioural treatment (e.g. coping or activity pacing) has been found to be beneficial for reducing fatigue and/or pain by stimulating a more regular pattern of activities and rest [45], and could play a role in optimising HR-QoL during and after rehabilitation. Activity pacing is a multifaceted coping strategy [46, 47], wherein people who perceive fatigue divide their energy and daily physical activities during the day. Activity pacing can be beneficial for: (1) people at risk of under activity and who are less aware of their energy distribution during the day [48] and (2) people at risk of over activity characterised by an uneven activity pattern consisting of high activity peaks followed by long periods of inactivity [49]. Health care professionals (e.g. sports counsellors or physiotherapists) may improve person-centred advice by motivational interviewing with a focus on activity pacing to reduce perceived fatigue and pain for sustained levels of high HR-QoL after rehabilitation.

Furthermore, we found that ‘not consuming alcohol’ distinguishes people in the recovery HR-QoL trajectory from people in the high HR-QoL trajectory before discharge. Also, we found confidence that people who do not smoke and/or drink alcohol were more likely to belong to the high HR-QoL trajectory compared to the moderate HR-QoL trajectory, but this finding was not statistically significant. This might be an indication of consequences of unhealthy lifestyle habits, like smoking and alcohol use, not sufficiently addressed during the rehabilitation treatment. More guidance, information and awareness related to general healthy lifestyle behaviours could potentially optimise rehabilitation programmes.

Finally, we did not find physical activity to be statistically significantly associated with HR-QoL trajectories. However, the direction of the association indicates that people who were more physically active before discharge from rehabilitation were more likely to follow the high HR-QoL trajectory compared to people in the moderate HR-QoL trajectory. This might imply that more physical activity is associated with higher HR-QoL, which supports previous literature [7, 9, 50, 51].

Lastly, no significant determinants were found to distinguish between the moderate versus recovery HR-QoL trajectories, probably because these trajectories had comparable HR-QoL scores at baseline. When we control for HR-QoL scores at baseline in the multiple binomial multivariable logistic regression analyses, we see that most significant determinants become non-significant. This implies that especially HR-QoL scores at baseline (the intercepts) of the moderate, high and recovery HR-QoL trajectories can be determined, while most personal-, disease- and lifestyle-related determinants are not able to differentiate between the course (slopes) of the HR-QoL trajectories up to 1 year after discharge from rehabilitation. Only perceived fatigue and pain are still significant determinants to distinguish between the moderate and high HR-QoL trajectories.

Some strengths and limitations of this study need to be addressed. HR-QoL scores (mean ± standard deviation) found in our cohort before discharge from rehabilitation (physical health: 36.2 ± 10.3; mental health: 40.3 ± 9.4) are comparable to a cohort of primary care patients with chronic diseases (physical health: 36.1 ± 10.8; mental health: 40.0 ± 10.8) [26]. However, HR-QoL scores in our sample are lower compared to people with type 2 diabetes (physical health: 43.5 ± 10.8; mental health: 44.8 ± 10.2) and people after total joint arthroplasty (physical health: 32.1 ± 8.1; mental health: 50.0 ± 9.2) [29].

In addition, we used LCGM models to unravel heterogeneity in HR-QoL after rehabilitation and to understand the underlying mechanisms for different subgroups in the population, which has some important advantages. First, this methodological technique categorises people based on their development pattern, a data-driven approach, instead of on a priori classification in theory-driven predefined groups [35, 52]. Furthermore, this LCGM approach categorises people in homogenous subgroups that represent different profiles of HR-QoL and subsequent health outcomes. This data-driven approach fits with the research design, an observational cohort study, but differs from the traditional way of summarising patient data into ‘the average patient’ [41]. An important point of discussion is the decision on the optimal number of classes, with respect to both the model fit criteria and clinical interpretation. Also, the sample size and the number of measurement occasions have been shown to influence the number and characteristics of the identified classes in the final model [53–56]. Choices made during the modelling process (e.g. model with the lowest BIC) may influence the interpretation of the models and subsequent implications. For example, the five-class quadratic model had a decline HR-QoL trajectory, but also a very small distinct strong recovery HR-QoL trajectory.

In addition, we used the two-step approach to evaluate the characteristics of the latent classes. In step one, we obtained the classes and assigned individuals to their most likely class. In step two, we assessed factors associated with class membership. These steps can also be combined into a one-step approach, where the extra variables are already included in the model during the (conditional) class formation process. Neither approach is right or wrong. The two-step approach for example ignores class assignment error, but does estimate the classes without covariates clouding the class formation [57, 58]. The one-step approach does incorporate the class assignment uncertainty, but covariates can influence the class formation process [57, 58]. Our posterior probabilities were relatively high and indicative of low membership error and the one-step approach does not always improve model fit.

Also, we used the RAND-12 questionnaire, which is not preferred over the extended, original RAND-36 questionnaire, nor over more disease-specific HR-QoL questionnaires. However, disease-specific questionnaires were not feasible in our heterogeneous cohort and the shorter RAND-12 version provided a solution to the problem to restrict the length of the questionnaire in the ReSpAct study in order to reduce the load for participants [24], which advances the commitment to participate in this longitudinal study.

Furthermore, we found differences between the sample included versus the sample excluded in the current study. Of interest are the acceptance of the disease, fatigue and smoking behaviour. These variables differed statistically significantly between the included and excluded sample as well as between the trajectories. Unfortunately, we were unable to determine the missing at random mechanism, because baseline variables of almost half of the excluded participants were missing.

### Implications for practice and research

More than one third of our sample obtained a relatively stable high HR-QoL, but more than half obtained moderate HR-QoL after participating in a person-centred physical activity promotion programme; the RSE programme. We found several modifiable disease-related factors to be important in determining HR-QoL, which emphasises the importance for optimising person-centred advice in focusing on fatigue and pain management and on better acceptance of the disability during rehabilitation. Also, the identified HR-QoL trajectories are not disease specific, which might imply a disease-overarching mechanism.

Furthermore, to make the LCGM modelling more transparent, the data, syntax and results are available in electronic supplementary material. Especially in latent trajectory studies, open communication is important due to the data-driven aspect of the analyses and the difficult choices made to find the optimal model fit. We would like to encourage other researchers in the field of latent trajectory studies, to provide open communication of their analyses and results, and to use the GRoLTS checklist [36] in reporting the analysis of the latent trajectory study. This will benefit comparison of the results in different study populations.

## Conclusion

This study identified three trajectories of HR-QoL after rehabilitation among a large heterogeneous cohort of people with a physical disability and/or chronic disease, of which there were two large stable trajectories (high and moderate), and one small intermediate trajectory (recovery). Our identified HR-QoL trajectories are comparable to HR-QoL trajectories identified in specific disease populations, which might indicate that HR-QoL trajectories are not disease specific. More than half of our sample obtained a relatively stable but moderate HR-QoL after rehabilitation, while 40.9% obtained a stable high HR-QoL. Membership of these HR-QoL trajectories were associated with a limited extend of personal-related factors (age and BMI), disease-related factors (perceived fatigue, perceived pain and acceptance of the disability) and one lifestyle-related factor (alcohol use) before discharge. The moderate HR-QoL trajectory may benefit from person-centred advice during rehabilitation on management of fatigue and pain (e.g. activity pacing), and the acceptance of the disability.

## Electronic supplementary material

Below is the link to the electronic supplementary material.

Supplementary file1 (CSV 36 kb)

Supplementary file2 (DOCX 2236 kb)
