# Supplementary material for: Trajectories of health-related quality of life among people with a physical disability and/or chronic disease during and after rehabilitation: a longitudinal cohort study
Source: Qual Life Res. 2020 Sep 28;30(1):67–80. doi: 10.1007/s11136-020-02647-7 (PMC7847859; doi:10.1007/s11136-020-02647-7)
Supplement: Supplementary file 2 — Supplementary file2 (DOCX 2236 kb) [file 11136_2020_2647_MOESM2_ESM.docx]

Seves BL, Hoekstra F, Hettinga FJ, Dekker R, van der Woude LHV, Hoekstra T. Trajectories of Health-related Quality of Life among people with a physical disability and/or chronic disease during and after rehabilitation: a longitudinal cohort study. Quality of Life Research.

**Supplemental information**

**Metric of time**

Timescores were specified according to the measurement waves in weeks of the Rehabilitation, Sports and Active Lifestyle (ReSpAct) study [1, 2]. Measurement times were at baseline (T0: 3-6 weeks before discharge) and 14 (T1), 33 (T2) and 52 (T3) weeks after discharge from rehabilitation (Fig. 1). For T0 we calculated the mean time point in weeks ((6-3)/2=4.5). Because the moment of discharge from rehabilitation is seen as week zero, measurement wave T0 becomes a negative value (-4.5) which is not practical in the Latent Class Growth Mixture modelling analyses in Mplus. Therefore we changed the measurement waves from -4.5 (T0), 14 (T1), 33 (T2) and 52 (T3) weeks to 0 (T0), 18.5 (T1), 37.5 (T2) and 56.5 (T3) weeks. Possible individual variance of time within the waves were not taken into account.


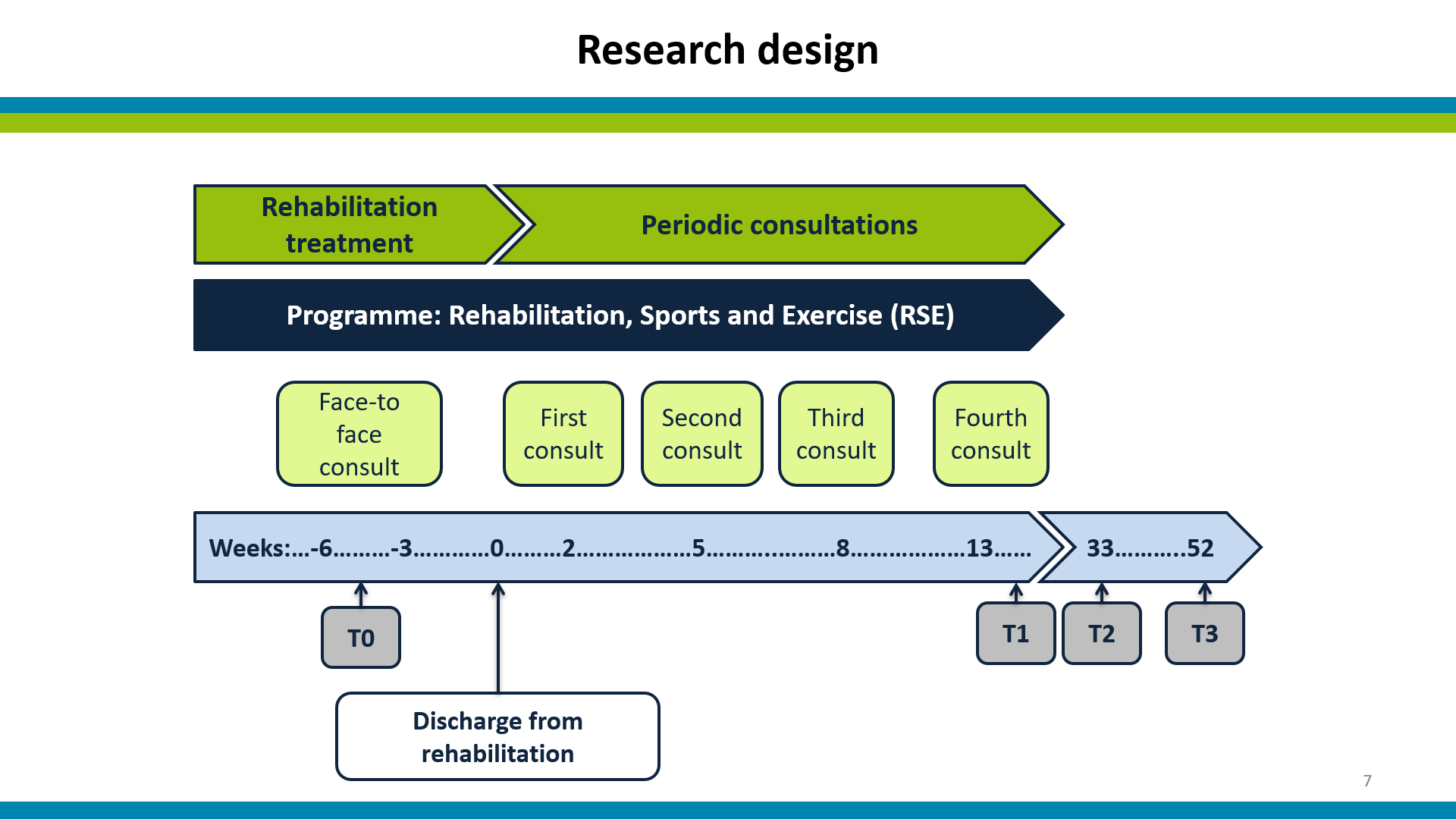

**Fig. 1 Schematic overview of the Rehabilitation, Sports and Exercise (RSE) programme with measurement moments in the Rehabilitation, Sports and Active lifestyle (ReSpAct) study.**

**Missing data**

The following missing data mechanism was used: the Expectation-Maximation algorithm [3]. This mechanism assumes missing at random. We found a trend towards attrition in the ReSpAct study. Participants included in the analysis were on average older and higher educated compared to participants not included in the analysis (Table 1).

*Table 1 Comparison of the included and excluded participants*

|  | Excluded, one available wave (N=324) | Included, two available waves (N=256) | Included, three available waves (N=316) | Included, four available waves (N=528) |
| --- | --- | --- | --- | --- |
|  | Mean ± SD or % (N) | | | |
| Gender (%female) | 59.6 (193) | 50.0 (128) | 56.0 (177) | 50.6 (267) |
| Age in years | 48.0 ± 13.2 | 50.0 ± 14.9 | 49.4 ± 13.7 | 52.4 ± 12.6 |
| BMI (kg/m^2^) | 27.9 ± 6.3 | 27.4 ± 6.0 | 27.4 ± 5.7 | 27.0 ± 5.1 |
| Education level (% high)^a^ | 21.6 (59) | 24.2 (54) | 24.9 (71) | 27.5 (145) |
| GHC T0 | 34.7 ± 8.8 | 35.6 ± 9.1 | 36.3 ± 9.0 | 38.2 ± 9.4 |
| GHC T1 | 36.3 ± 8.5 | 38.5 ± 10.1 | 38.5 ± 9.9 | 39.4 ± 10.2 |
| GHC T2 | 37.4 ± 9.7 | 39.3 ± 10.9 | 38.3 ± 10.3 | 40.6 ± 10.4 |
| GHC T3 | 37.6 ± 10.9 | 37.6 ± 10.3 | 38.2 ± 10.5 | 41.0 ± 10.7 |

^a^ Completed applied University or higher
Body Mass Index (BMI); General Health Composite (GHC); Not Applicable (NA)

**Description of the data set**

Electronic supplemental material 1 (ESM1) includes the data set for the Latent Class Growth Mixture Modelling analyses to identify trajectories of Health-related Quality of Life. The data set consists of five variables for all 1100 participants: (1) participant number [ID], (2) general health score at T0 [GHC_T0], (3), general health score at T1 [GHC _T1], (4) general health score at T2 [GHC _T2], and (5) general health score at T3 [GHC _T3]. Missing values are encoded with 9999.

**Mplus syntax of the Latent Class Growth Mixture modelling**

PLOT:

TYPE IS PLOT3;

SERIES IS GHC_T0 GHC_T1 GHC_T2 GHC_T3 (*);

MODEL:

%OVERALL%

I S Q | GHC_T0@0 GHC_T1@18.5 GHC_T2@37.5 GHC_T3@56.5;

%CLASS#1%

S@0;

Q@0;

%CLASS#2%

S@0;

Q@0;

%CLASS#3%

S@0;

Q@0;

**SPSS syntax of the multiple binomial multivariable logistic regression analyses**

*Compare class 2 (moderate HR-QoL) and class 3 (high HR-QoL).

*select classes.

USE ALL.

COMPUTE filter_$=(Class_quadratic3 = 2 | Class_quadratic3 = 3).

VARIABLE LABELS filter_$ 'Class_quadratic3 = 2 | Class_quadratic3 = 3 (FILTER)'.

VALUE LABELS filter_$ 0 'Not Selected' 1 'Selected'.

FORMATS filter_$ (f1.0).

FILTER BY filter_$.

EXECUTE.

*Perform logistic regression analyses.

LOGISTIC REGRESSION VARIABLES Class_quadratic3

/METHOD=ENTER Gender_T0 ZAge_T0 ZBMI_T0 EducationLevel_T0

/METHOD=ENTER Diseasegroup_T0 AcceptanceDis_T0 Comorbidities_T0 ZFSS_T0 Pain_T0

/METHOD=ENTER Smoke_T0 Alcohol_T0 ZPA_totmwk_T0 Sports_T0

/CONTRAST (Gender_T0)=Indicator(1)

/CONTRAST (EducationLevel_T0)=Indicator(1)

/CONTRAST (Diseasegroup_T0)=Simple(1)

/CONTRAST (AcceptanceDis_T0)=Indicator(1)

/CONTRAST (Comorbidities_T0)=Indicator(1)

/CONTRAST (Pain_T0)=Indicator(1)

/CONTRAST (Smoke_T0)=Indicator(1)

/CONTRAST (Alcohol_T0)=Indicator(1)

/CONTRAST (Sports_T0)=Indicator

/CLASSPLOT

/PRINT=GOODFIT CORR ITER(1) CI(95)

/CRITERIA=PIN(0.05) POUT(0.10) ITERATE(20) CUT(0.5).

*Same for comparison of the other latent classes.

**Supplemental figures of the final model**


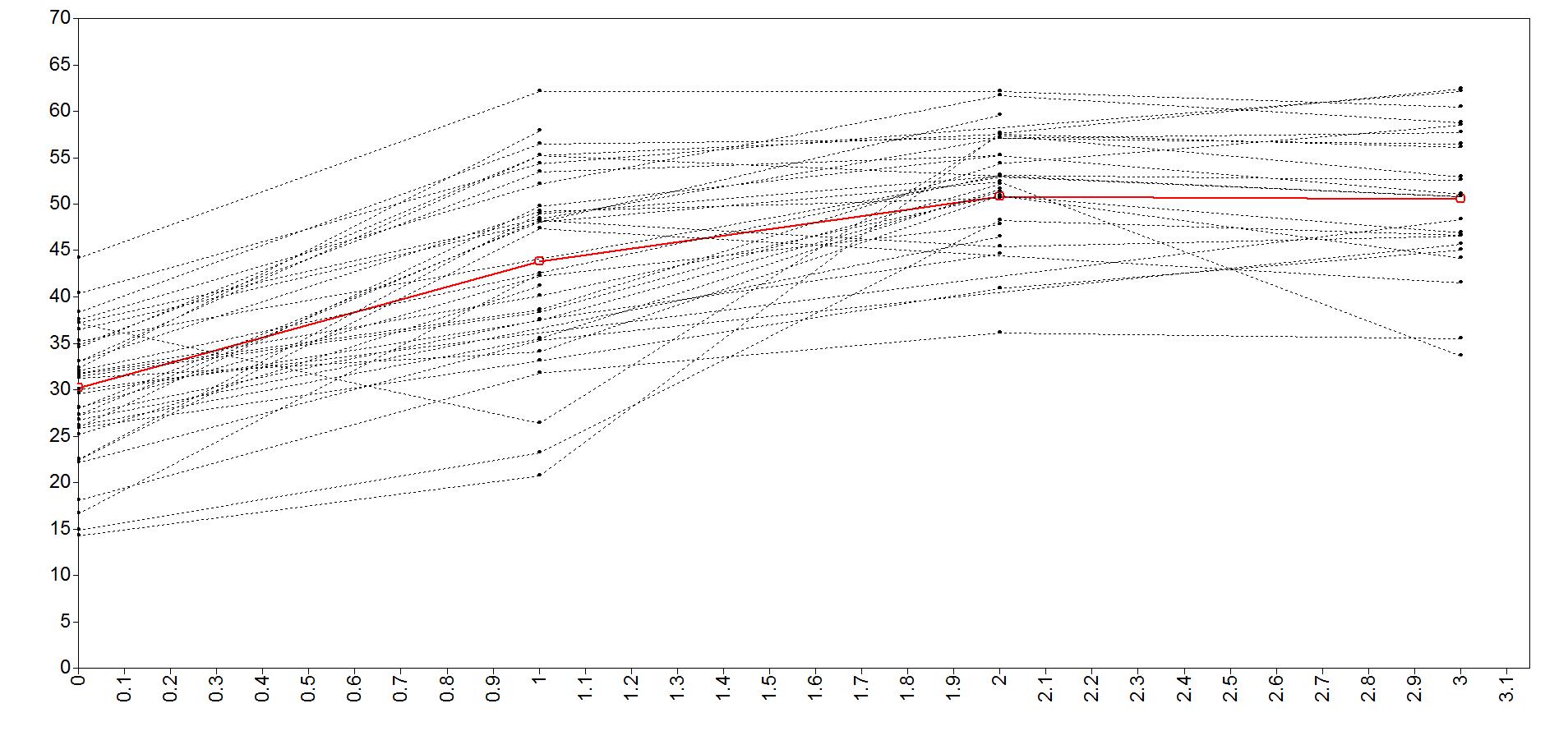


**Fig. 2 The estimated mean with observed individual trajectories of the first latent class of the final model (N=36).**


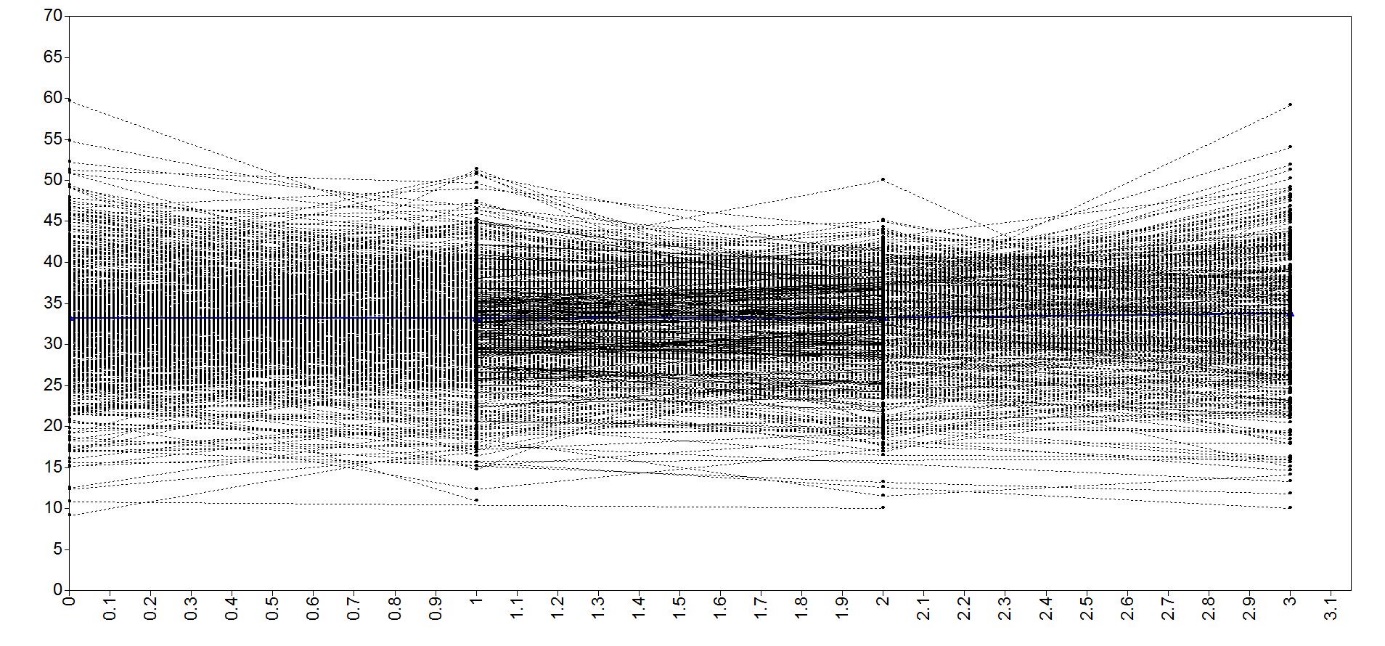


**Fig. 3 The estimated mean with observed individual trajectories of the second latent class of the final model (N=635).**


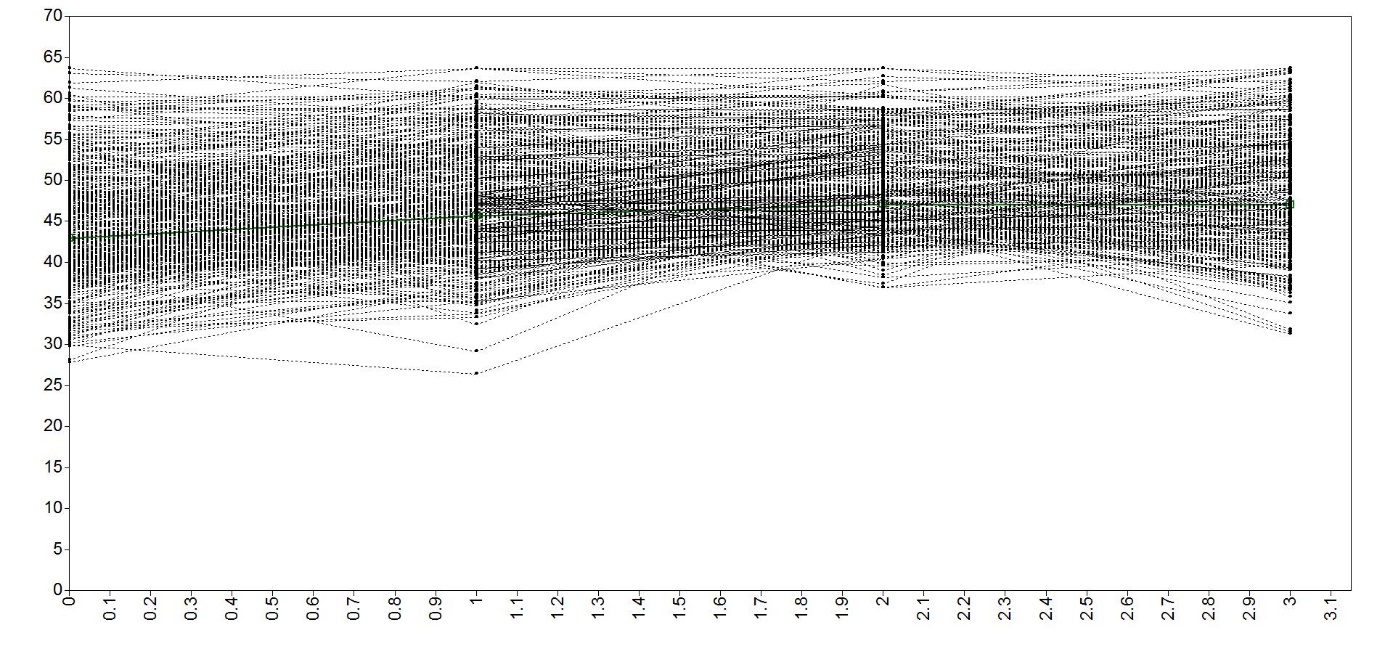


**Fig. 4 The estimated mean with observed individual trajectories of the third latent class of the final model (N=429).**


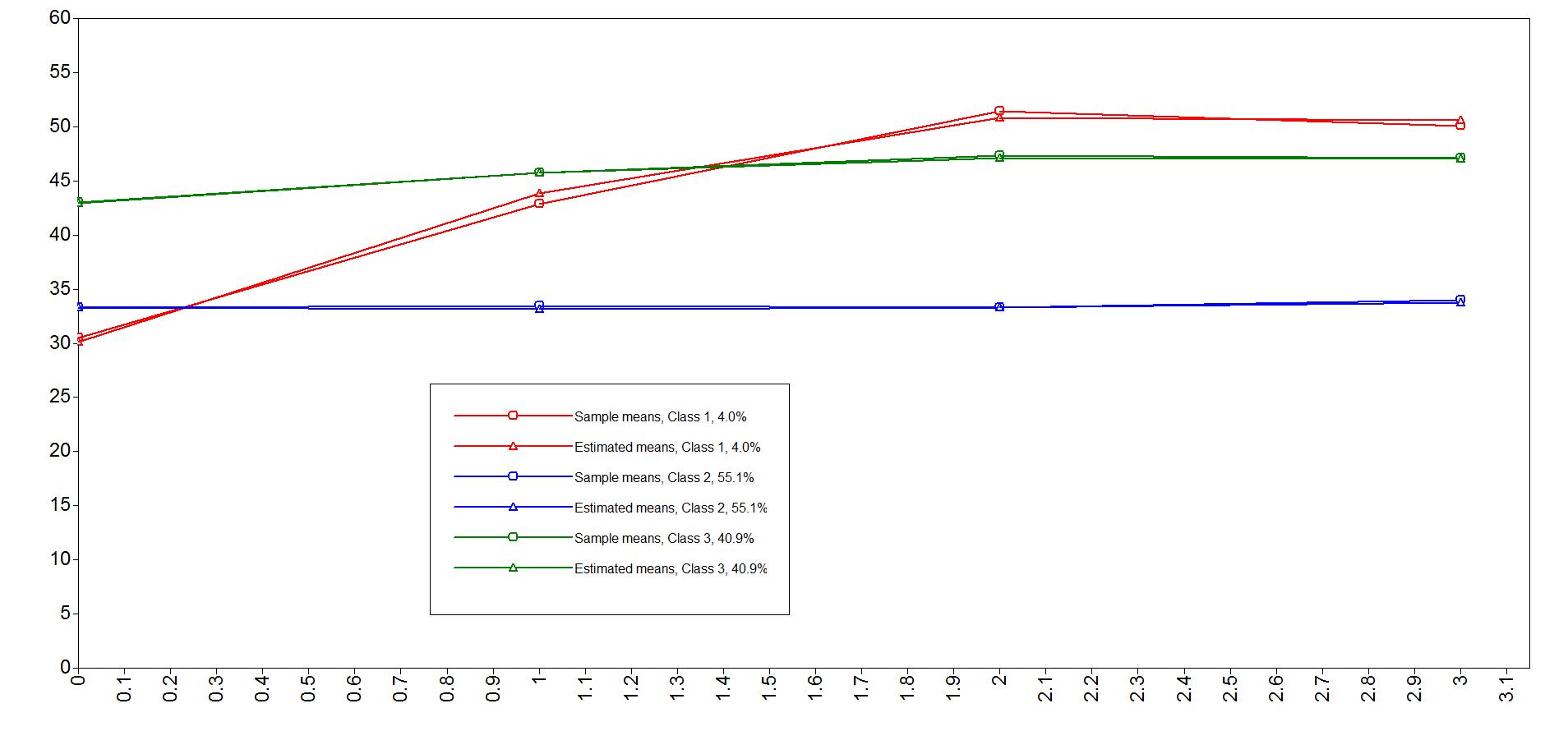


**Fig. 5 The observed and estimated mean trajectories of the final model: three-class quadratic model.**

**Supplemental figures of the alternative models**


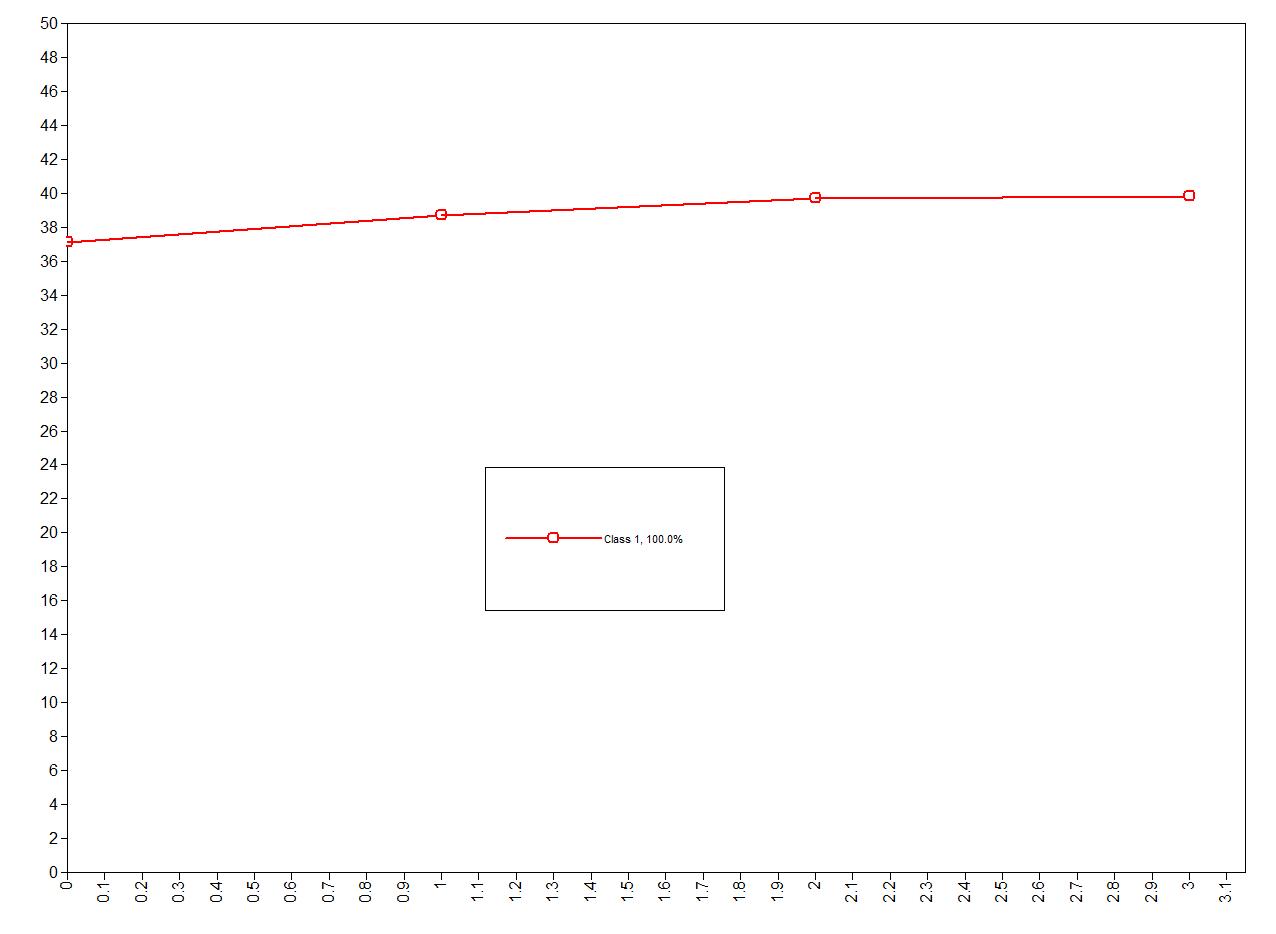


**Fig. 6 The estimated mean trajectory of the one-class quadratic model**


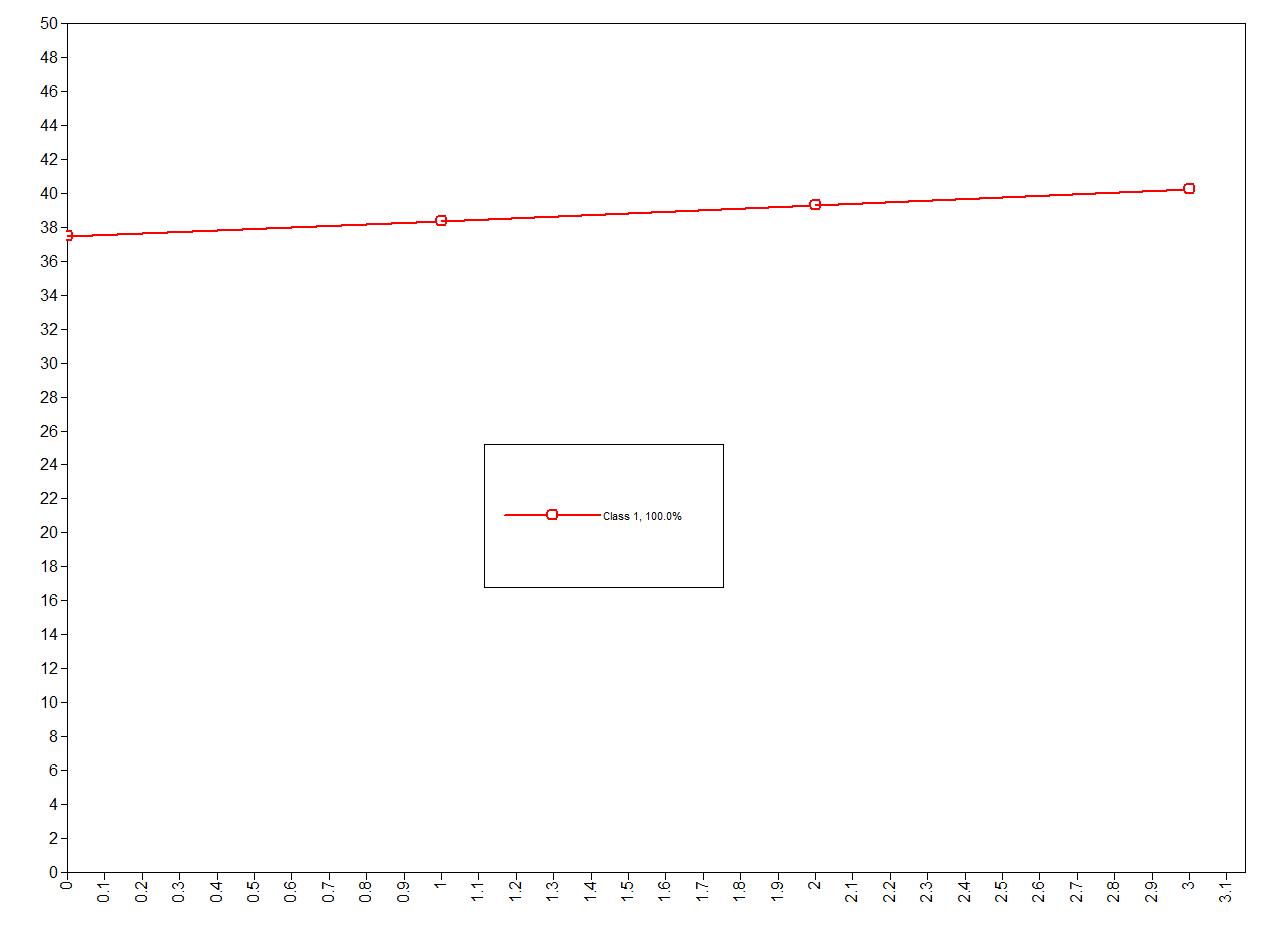


**Fig. 7 The estimated mean trajectory of the one-class linear model**


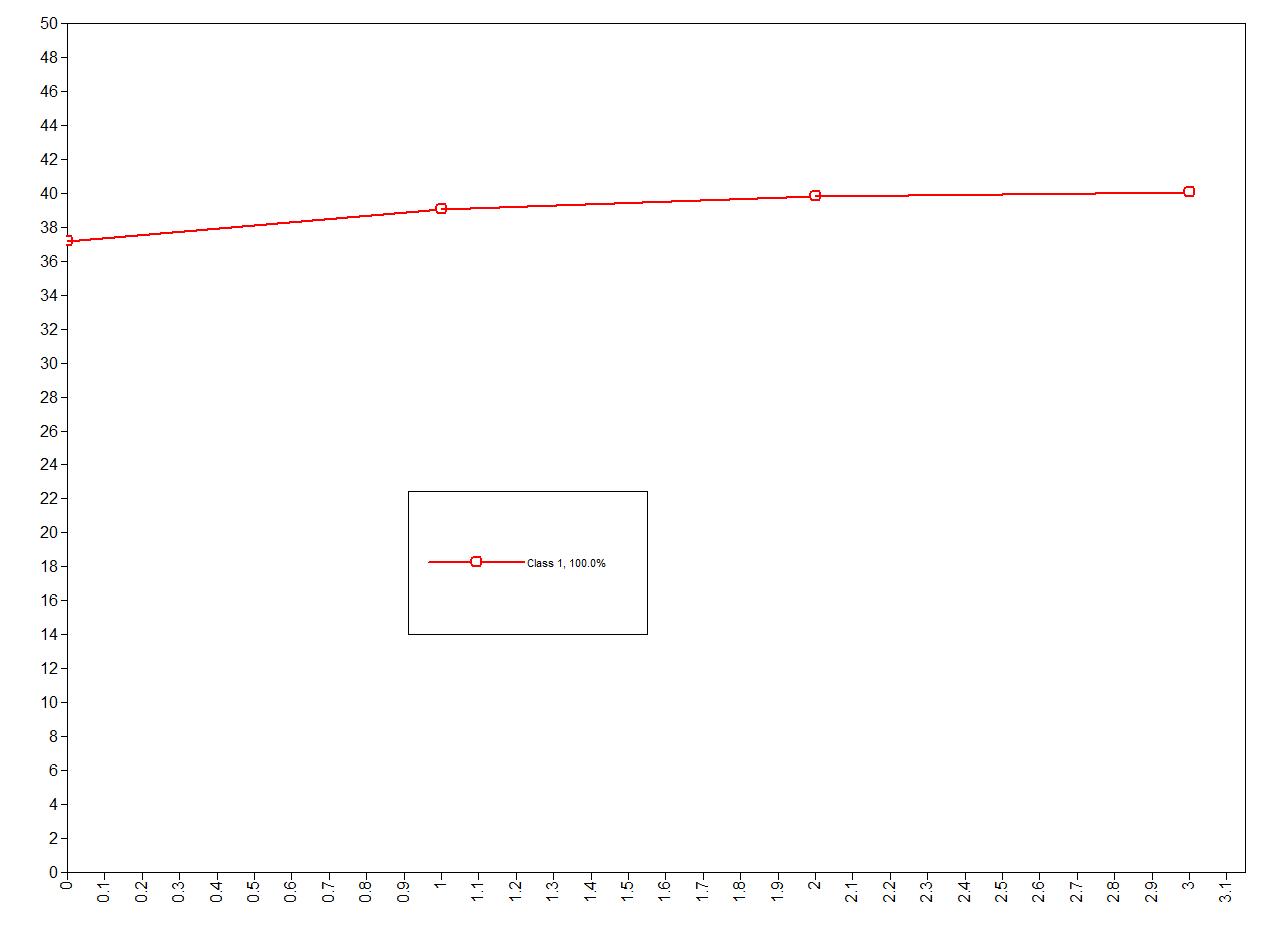


**Fig. 8 The estimated mean trajectory of the one-class latent class analyses model**


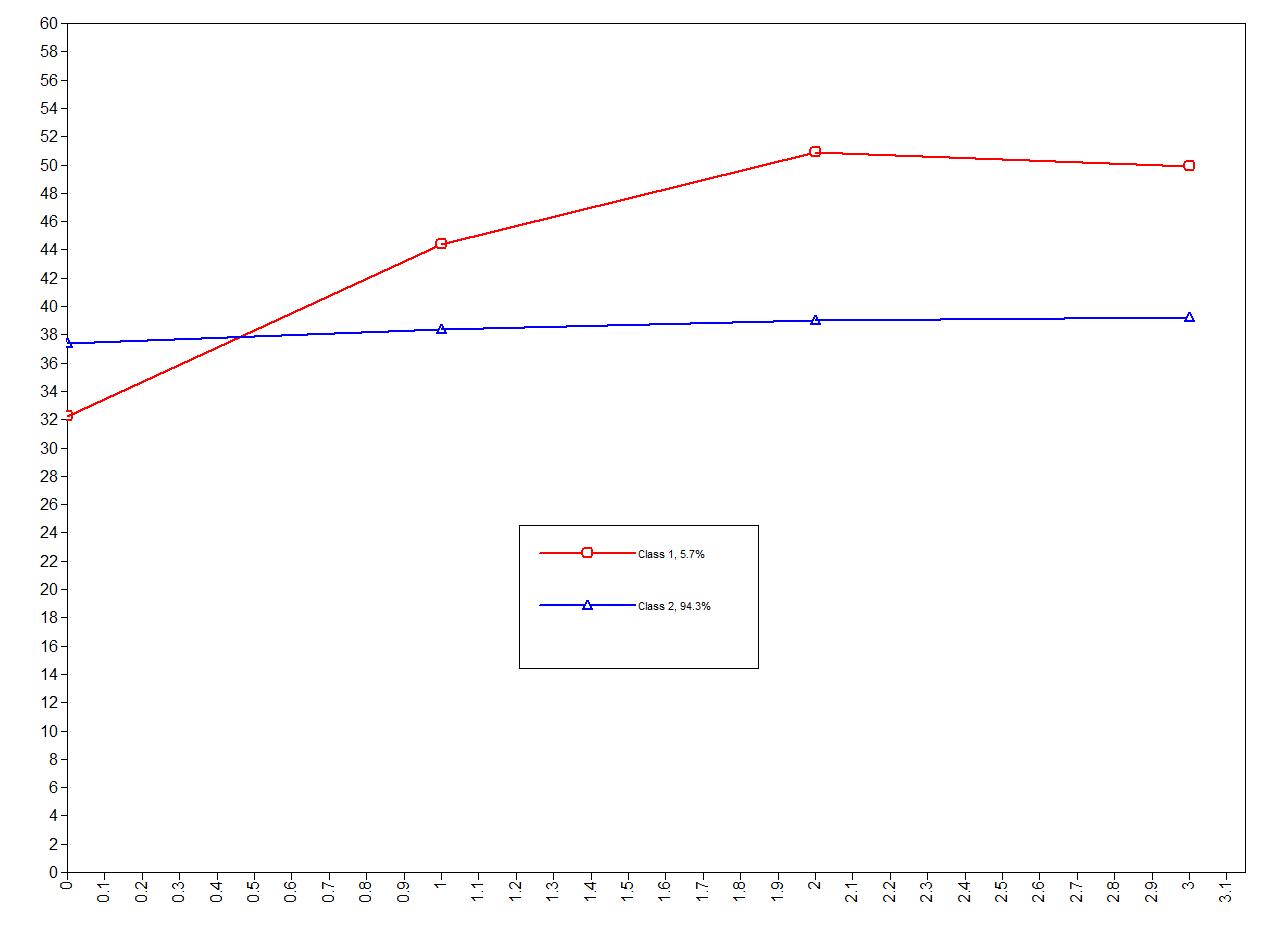


**Fig. 9 The estimated mean trajectories of the two-class quadratic model**


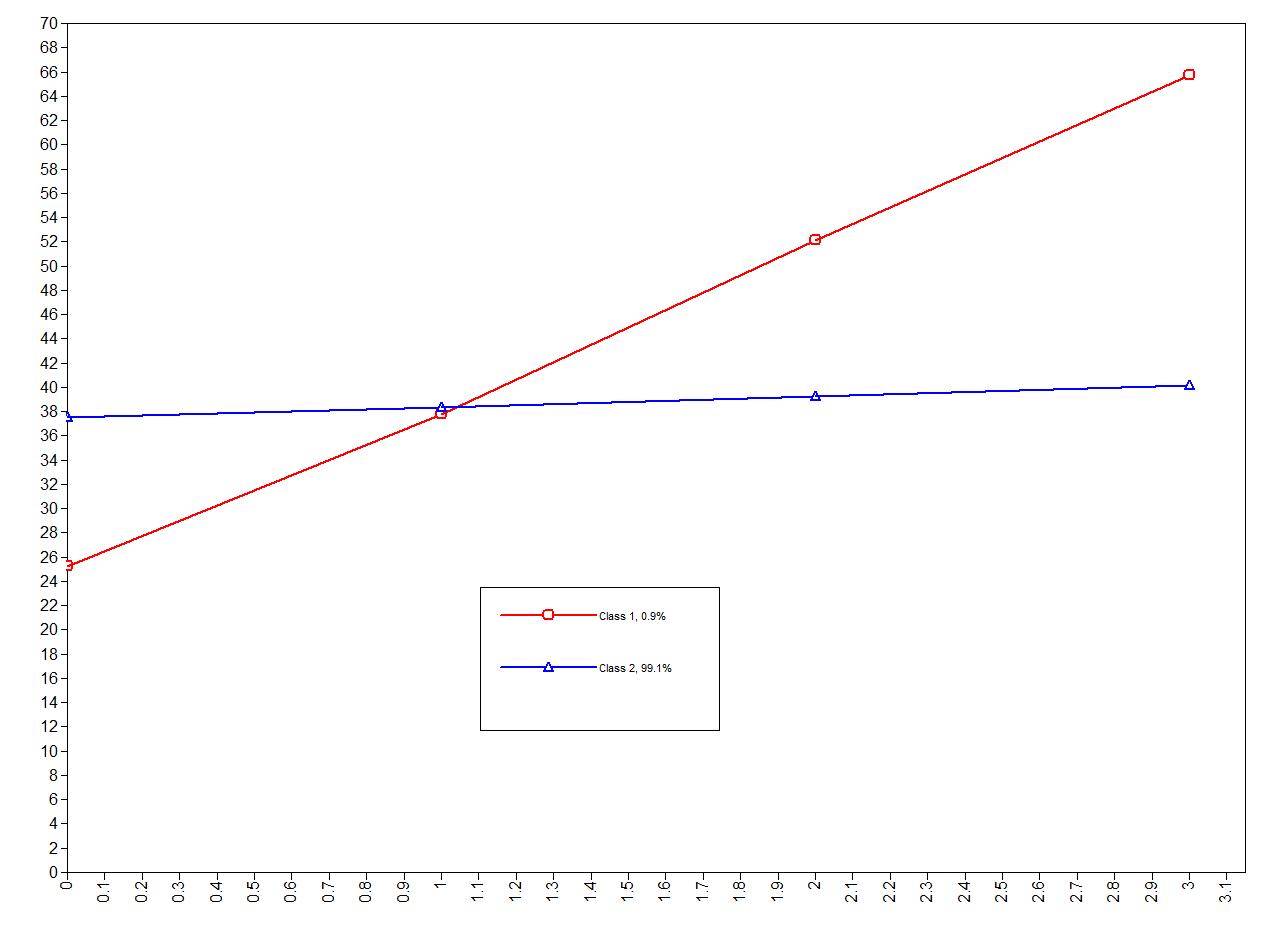


**Fig. 10 The estimated mean trajectories of the two-class linear model**


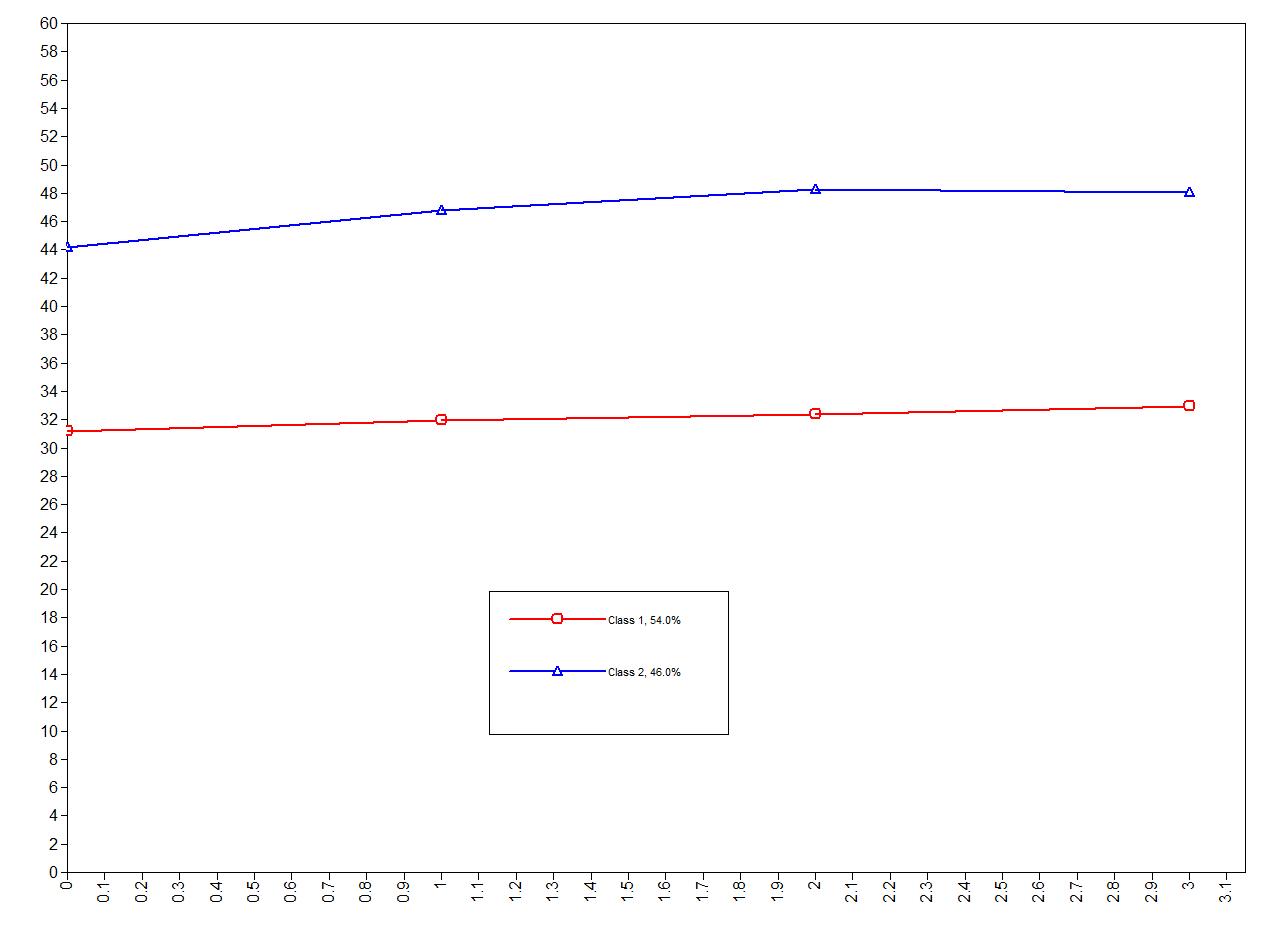


**Fig. 11 The estimated mean trajectories of the two-class latent class analyses model**


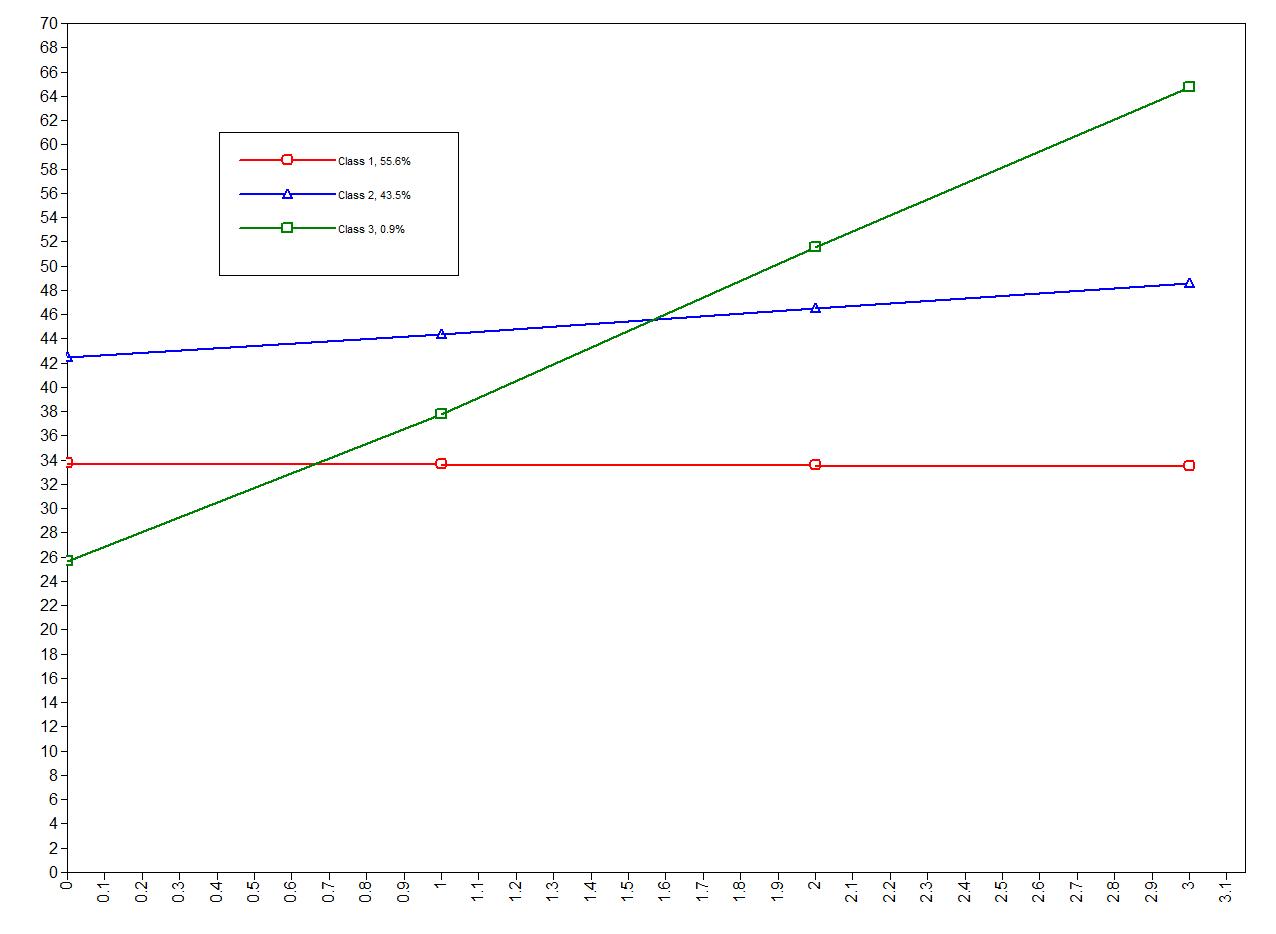


**Fig. 12 The estimated mean trajectories of the three-class linear model**


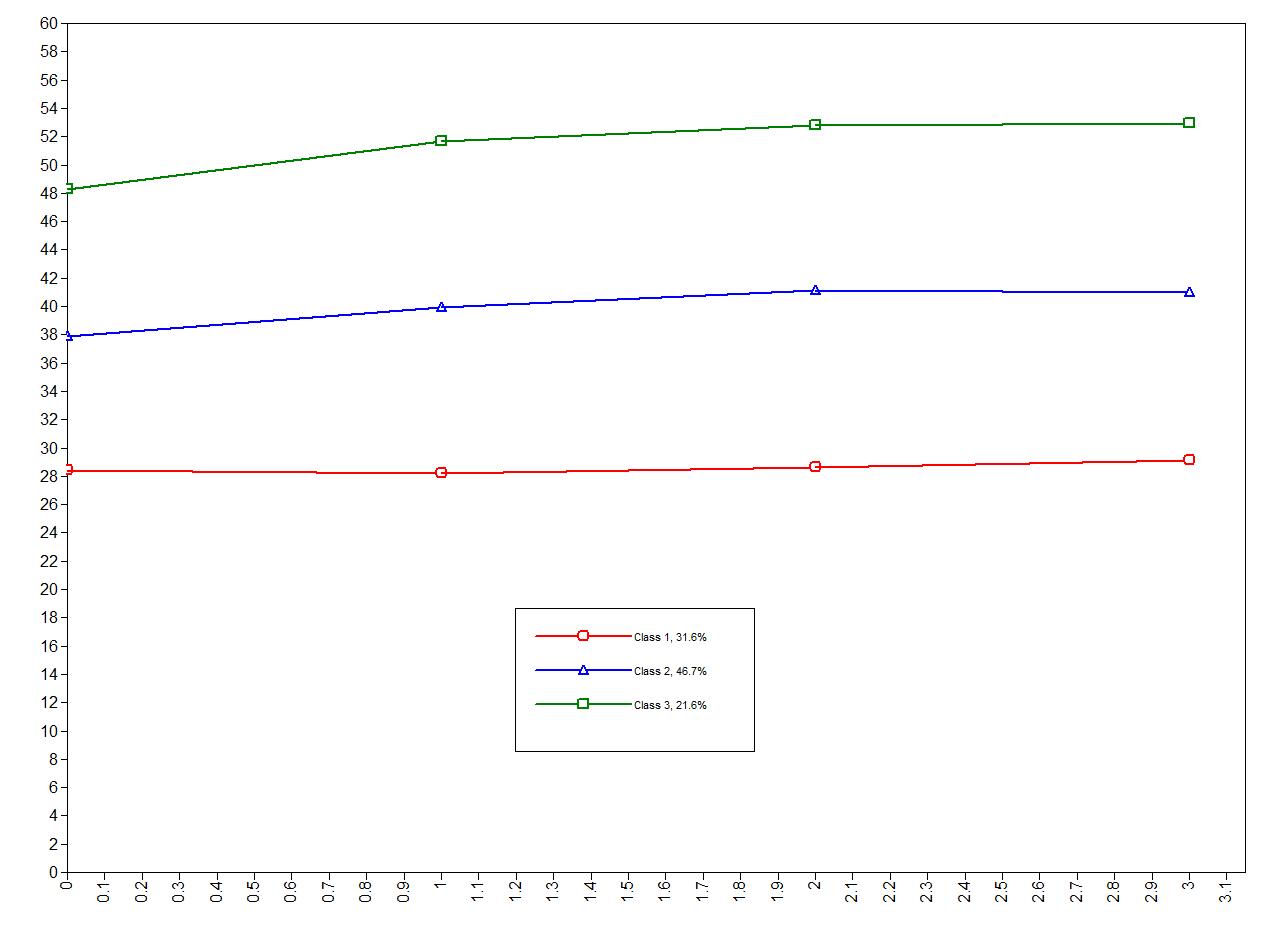


**Fig. 13 The estimated mean trajectories of the three-class latent class analyses model**


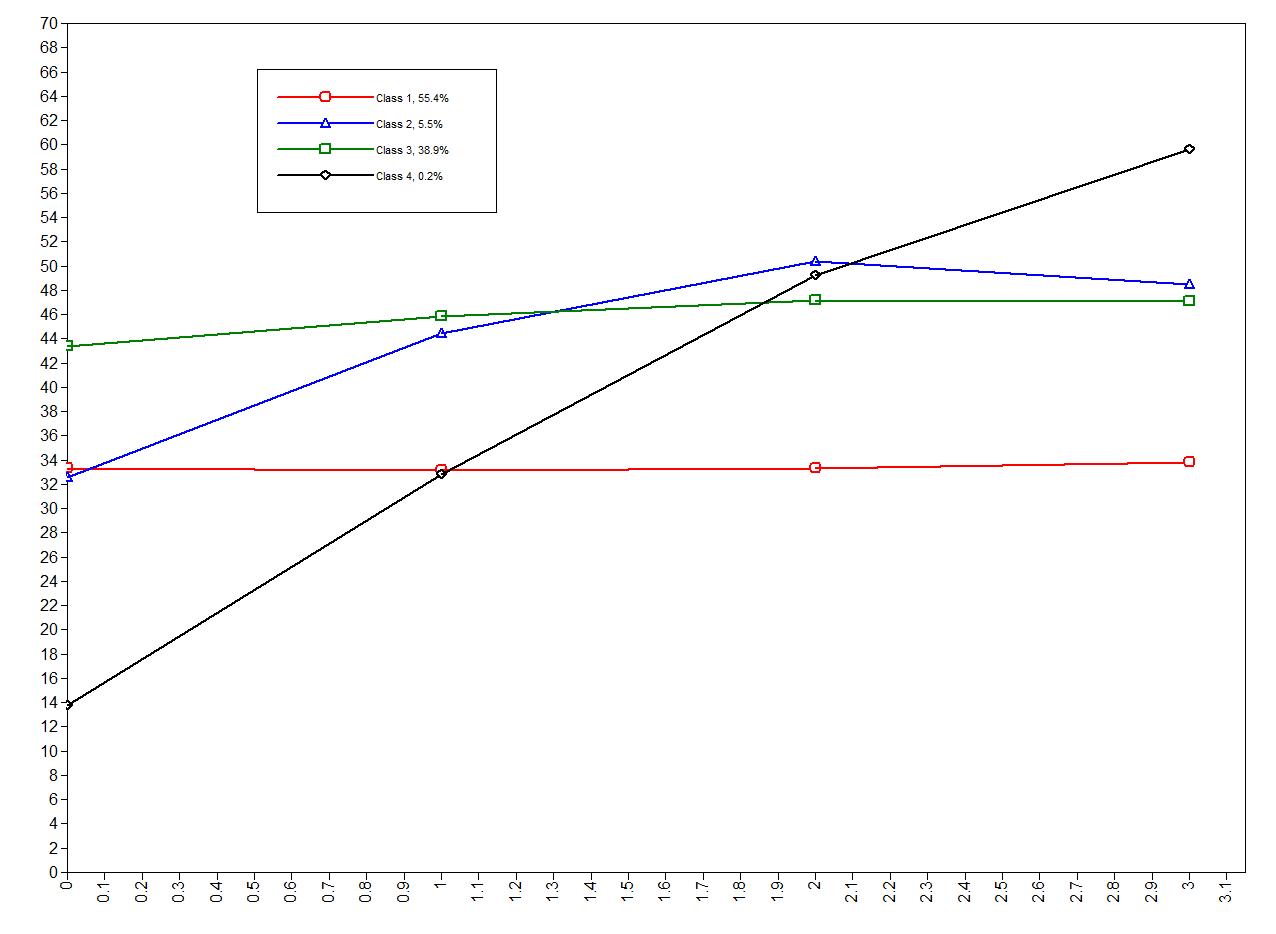


**Fig. 14 The estimated mean trajectories of the four-class quadratic model**


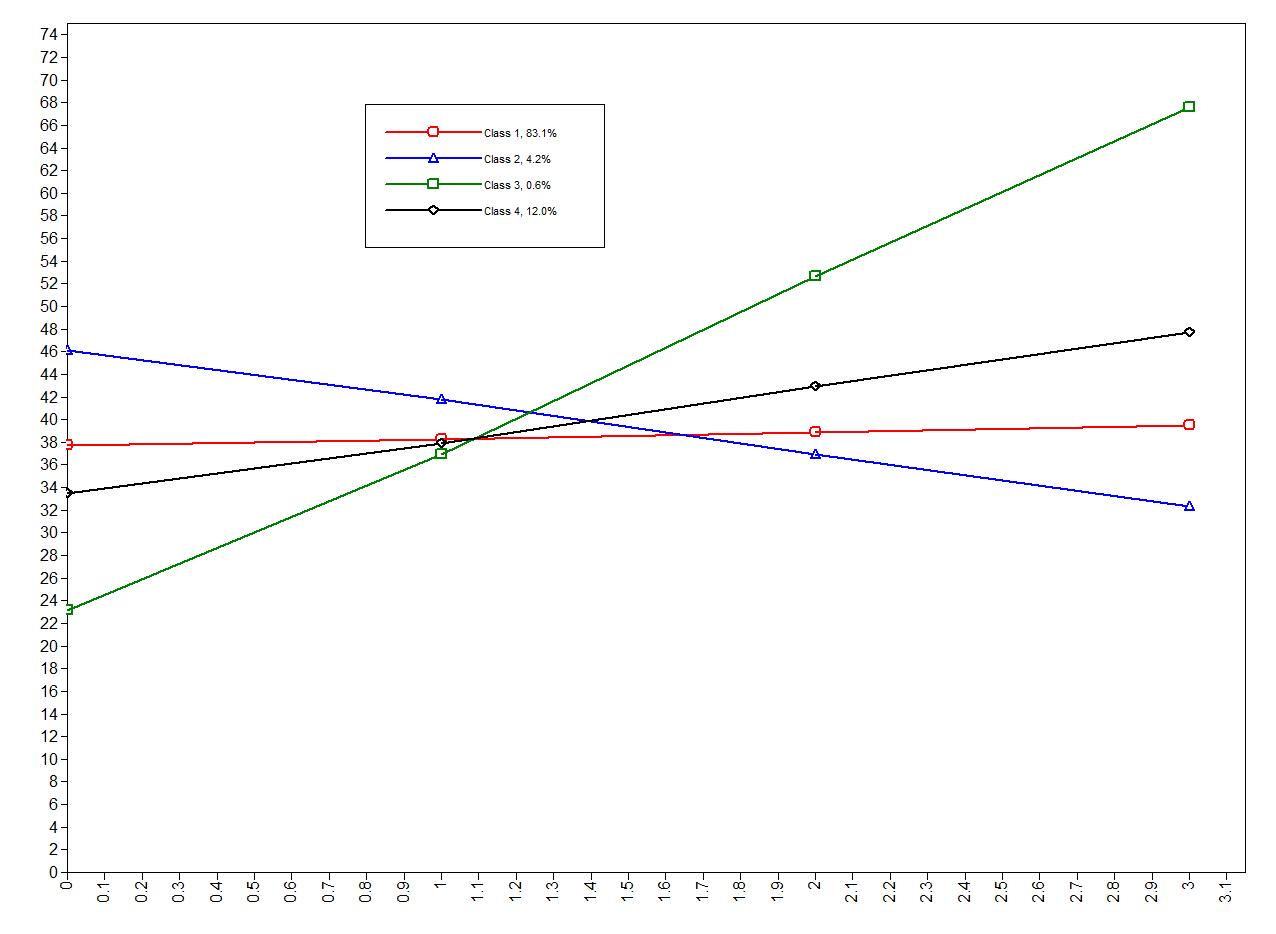


**Fig. 15 The estimated mean trajectories of the four-class linear model**


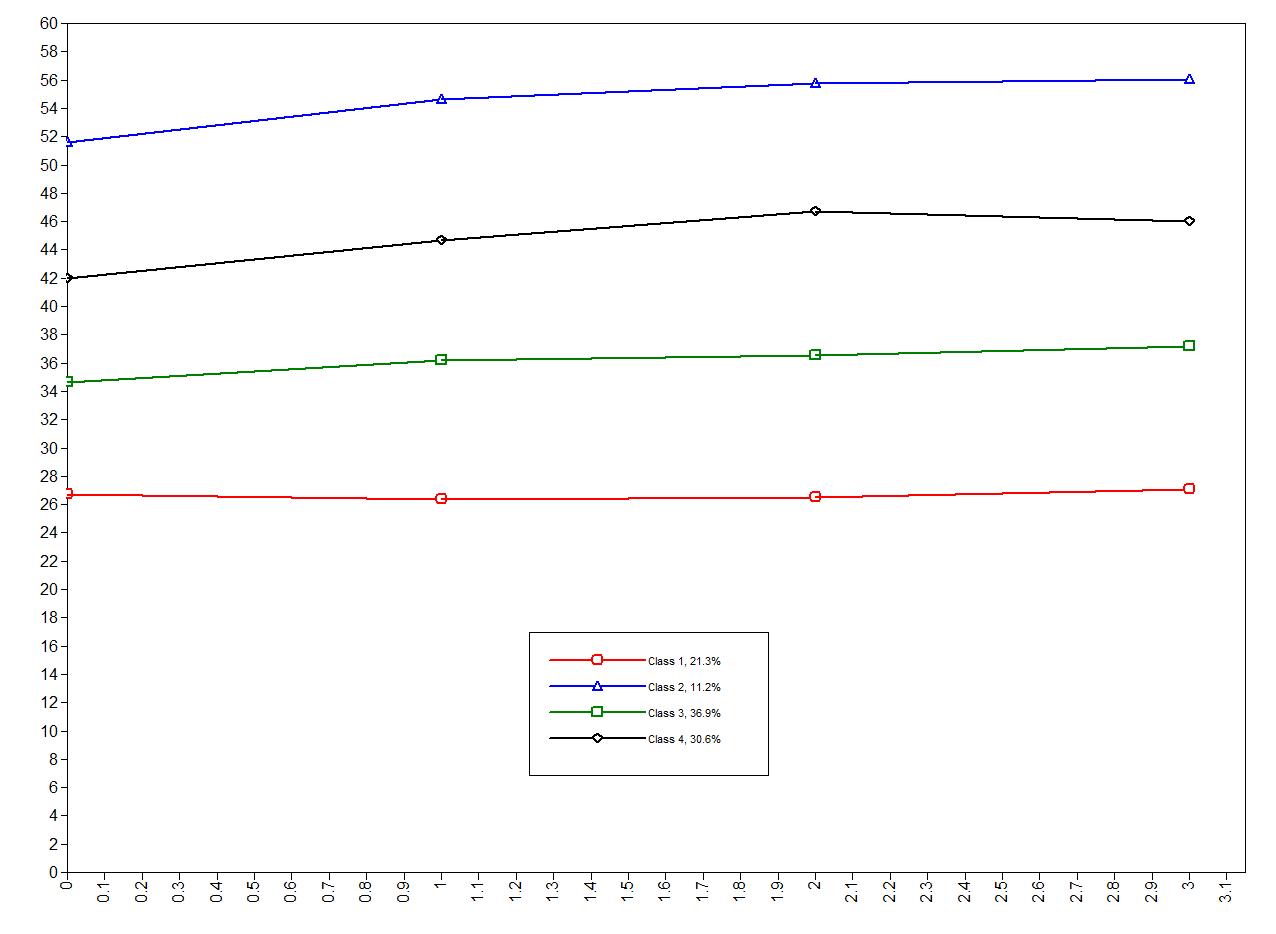


**Fig. 16 The estimated mean trajectories of the four-class latent class analyses model**


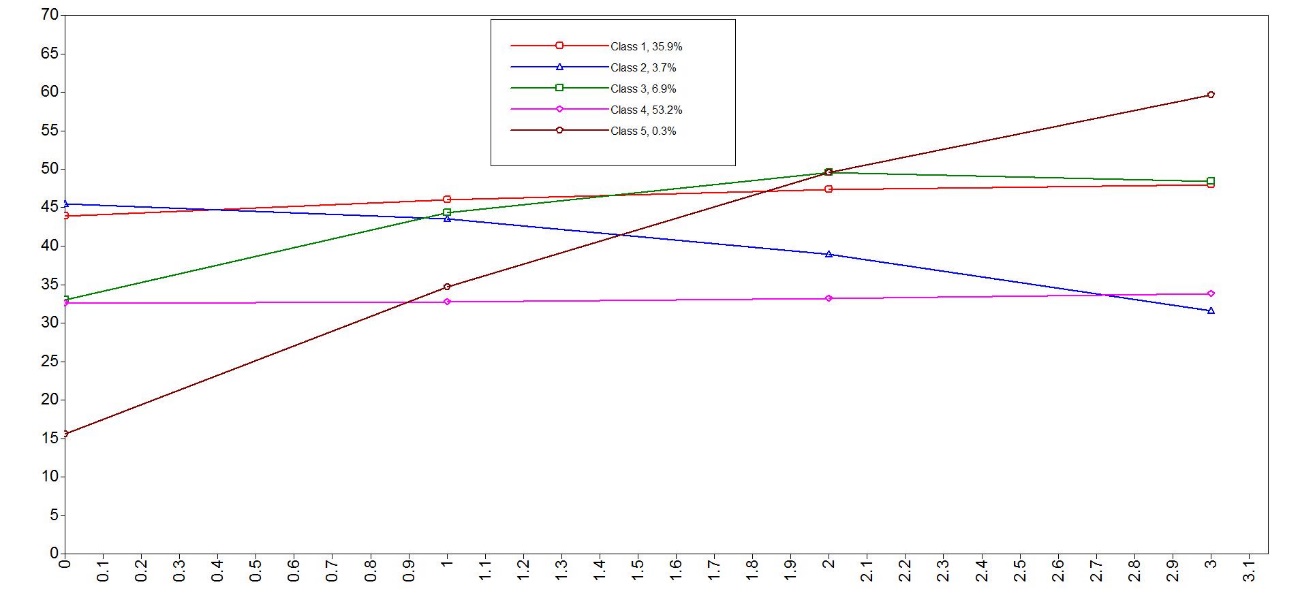


**Fig. 17 The estimated mean trajectories of the five-class quadratic model**


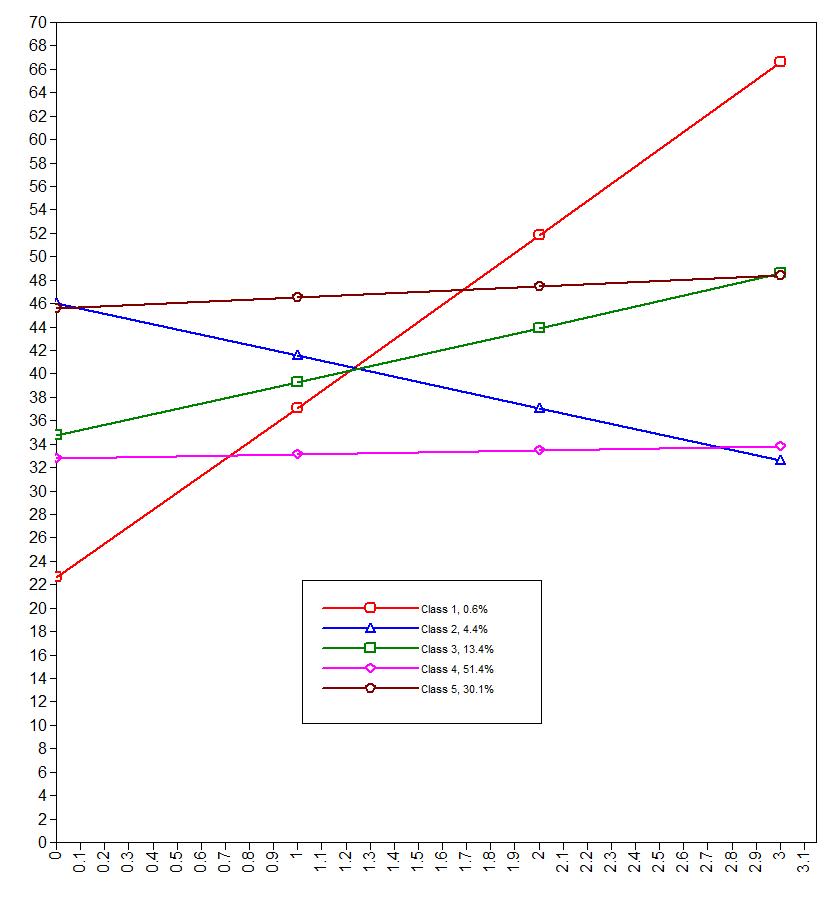


**Fig. 18 The estimated mean trajectories of the five-class linear model**


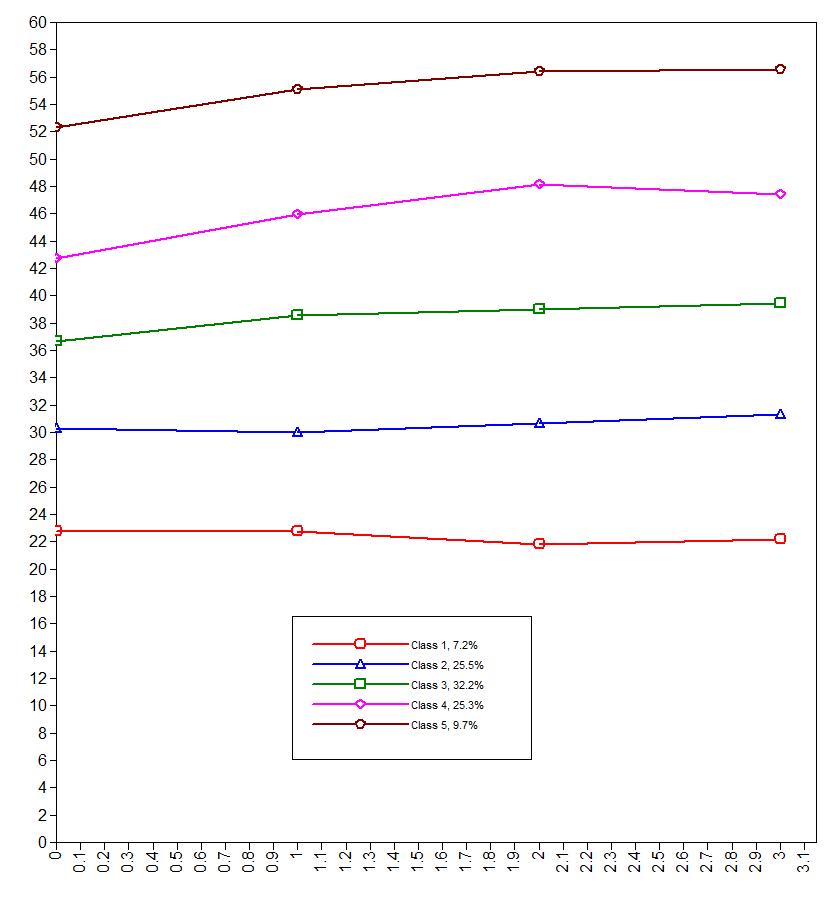


**Fig. 19 The estimated mean trajectories of the five-class latent class analyses model**


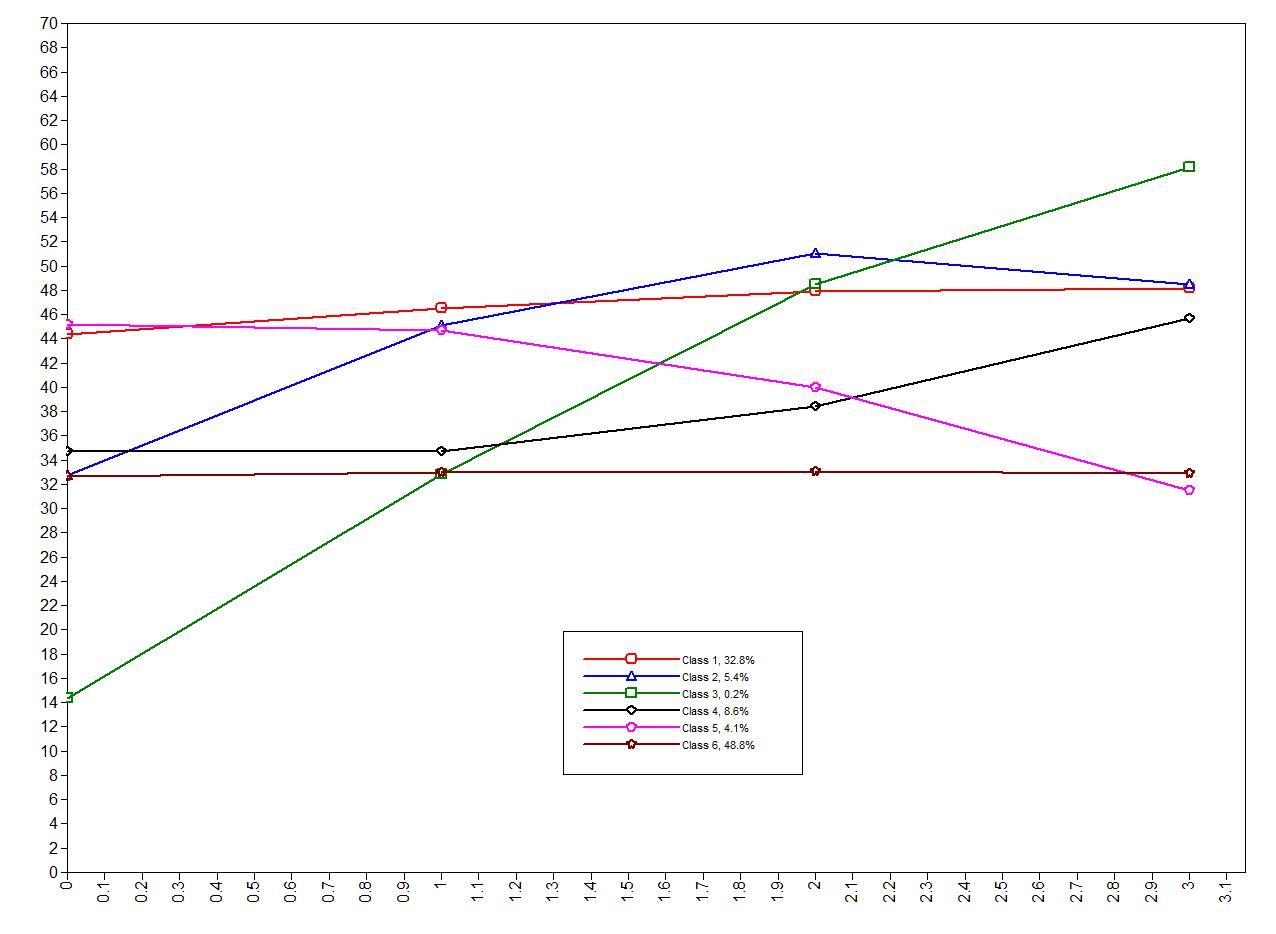


**Fig. 20 The estimated mean trajectories of the six-class quadratic model**


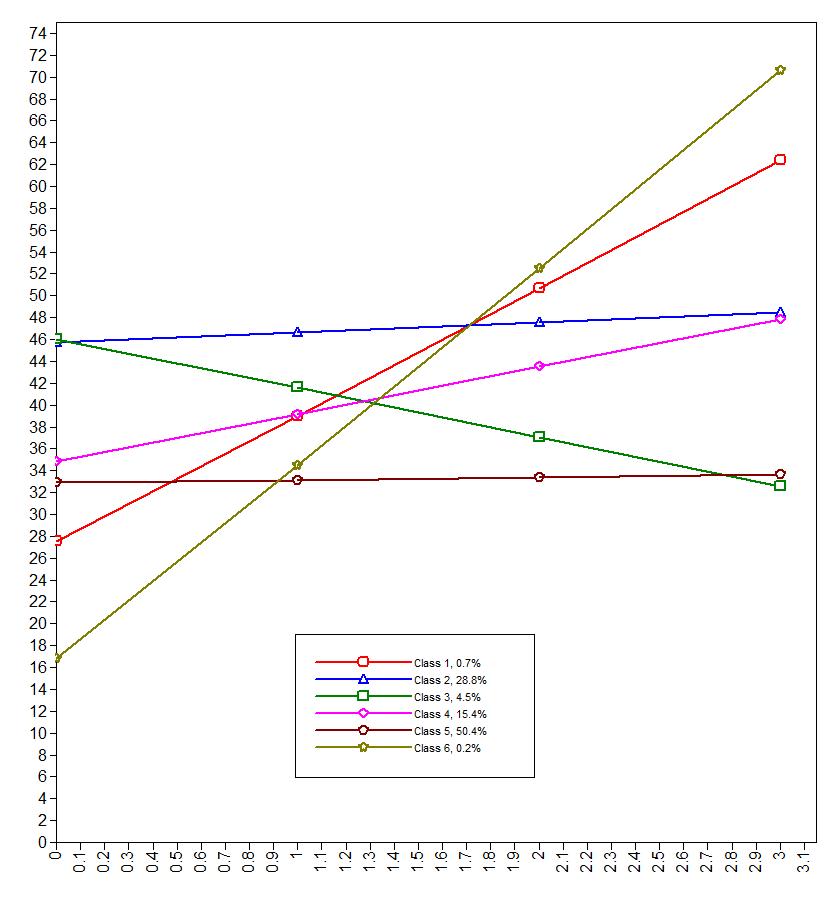


**Fig. 21 The estimated mean trajectories of the six-class linear model**


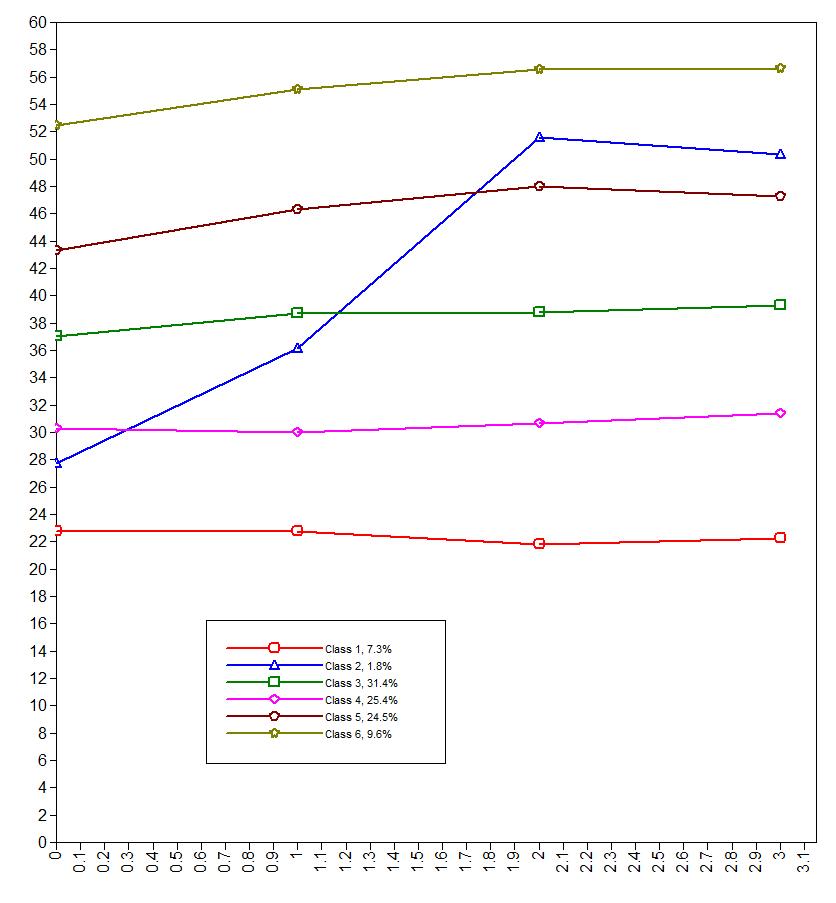


**Fig. 22 The estimated mean trajectories of the six-class latent class analyses model**

**References**

1. Hoekstra F, Alingh RA, van der Schans CP, Hettinga FJ, Duijf M, Dekker R, et al. Design of a process evaluation of the implementation of a physical activity and sports stimulation programme in dutch rehabilitation setting: ReSpAct. Implement Sci. 2014 Sep 20;9:127,014-0127-7.

2. Alingh RA, Hoekstra F, van der Schans CP, Hettinga FJ, Dekker R, van der Woude LH. Protocol of a longitudinal cohort study on physical activity behaviour in physically disabled patients participating in a rehabilitation counselling programme: ReSpAct. BMJ Open. 2015 Jan 29;5(1):e007591,2015-007591.

3. McLachlan G, Krishnan T. The EM algorithm and extensions. Hoboken, NJ: John Wiley & Sons, Inc; 2008.
